# *catena*-Poly[[tetra­kis­(3,5-dimethyl-1*H*-pyrazole-κ*N* ^2^)copper(II)]-μ_2_-sulfato-κ^2^ *O*:*O*′]: crystal structure and Hirshfeld surface analysis of a Cu^II^ coordination polymer

**DOI:** 10.1107/S2056989022002894

**Published:** 2022-03-24

**Authors:** Oleksandr S. Vynohradov, Artur Dovzhik, Vadim A. Pavlenko, Dina D. Naumova, Irina A. Golenya, Sergiu Shova

**Affiliations:** a Department of Chemistry, Taras Shevchenko National University of Kyiv, Volodymyrska str. 64/13, 01601 Kyiv, Ukraine; b "Poni Petru" Institute of Macromolecular Chemistry, Aleea Gr. Ghica, Voda 41A, 700487 Iasi, Romania

**Keywords:** copper, copper complexes, pyrazole, coordination polymer, Hirshfeld surface analysis, supra­molecular assembly, direct synthesis, oxidative dissolution, crystal structure

## Abstract

The title coordination polymer, [Cu(SO_4_)(C_5_H_8_N_2_)_4_]_*n*_, was synthesized using a one-pot reaction of copper powder, anhydrous copper(II) sulfate and 3,5-dimethyl-1*H*-pyrazole (Hdmpz) in aceto­nitrile under ambient conditions. The asymmetric unit can be described as a chain consisting of four [Cu(SO_4_)(Hdmpz)_4_] formula units that are connected to each other by a μ_2_-sulfato-bridged ligand. The octa­hedral coordination geometry (O_2_N_4_) of all copper atoms is realized by coordination of four pyrazole ligands and two sulfate ligands. Four pyridine-like N atoms of the pyrazole ligands occupy the equatorial positions, while two oxygen atoms of two sulfate ligands are in axial positions. As a result of the sulfate ligand rotation, there is a pairwise alternation of terminal O atoms (which are not involved in coordination to the copper atom) of the SO_4_ tetra­hedra. The Cu⋯Cu distances within one asymmetric unit are in the range 7.0842 (12)–7.1554 (12) Å. The crystal structure is built up from polymeric chains packed in a parallel manner along the *b*-axis direction. Hirshfeld surface analysis suggests that the most important contributions to the surface contacts are from H⋯H (74.7%), H⋯O/O⋯H (14.8%) and H⋯C/C⋯H (8.2%) inter­actions.

## Abstract 2

The title coordination pyrazole-containing coordination polymer, was synthesized using a one-pot reaction of copper powder, anhydrous copper(II) sulfate and 3,5-dimethyl-1*H*-pyrazole (Hdmpz) in aceto­nitrile under ambient conditions. The crystal structure is built up from packed parallel polymeric chains, which are stabilized by an extensive hydrogen-bond network, which forms with the participation of bridging sulfate ligands.

## Chemical context

The synthesis, structure and properties of metal complexes, including coordination polymers, is an important area of chemical research. The nature of the anion, which is part of a coordination compound, is one of several factors that has a great influence on the final structural topology of the complexes (Mondal *et al.*, 2009 ▸; Mahmoudi *et al.*, 2007 ▸; Kwak *et al.*, 2008 ▸; Balić *et al.*, 2018 ▸). A large number of coordination compounds have been synthesized and studied due to the development of supra­molecular chemistry and the study of self-assembly of metal complexes with organic mol­ecules, such as pyrazoles. These mol­ecules have long been recognized as useful ligands for studying transition-metal coordination chemistry (Mihailov *et al.*, 1974 ▸; Nicholls *et al.*, 1971 ▸; Reedijk, 1971 ▸, 1970*a* ▸,*b* ▸; Reedijk & Smit, 1971 ▸; Reedijk *et al.*, 1971 ▸; Singh *et al.*, 1973 ▸; ten Hoedt *et al.*, 1982 ▸). Pyrazole-based ligands are used to construct supra­molecular architectures due to the presence of a pyrrole NH group in the pyrazole ring, which is not necessarily coordinated by a metal atom, but may act as a donor of hydrogen bonds. In addition, substituents on the pyrazole ring can also be involved in hydrogen-bond inter­actions. These facts are very important because there is a noticeable influence of hydrogen bonding on coordination compound assembly (Di Nicola *et al.*, 2007 ▸; Brewer *et al.*, 2020 ▸; Burrows *et al.*, 2011 ▸). The crystal packing of coordination polymers also depends on the different solvents employed, although not necessarily incorporating the solvents as crystallization mol­ecules (Di Nicola *et al.*, 2014 ▸). Reaction of a metal salt with an organic ligand is a popular way for the synthesis of coordination compounds, including metal coord­ination polymers (Gogoi *et al.*, 2019 ▸; Shen *et al.*, 2004 ▸), but there are many types of coordination compounds and the methods of synthesis are varied (House *et al.*, 2016 ▸). In this article we report the preparation of the coordination polymer *catena*-poly[[tetra­kis­(3,5-dimethyl-1*H*-pyrazole-κ*N* ^2^)copper(II)]-μ_2_-sulfato-κ^2^ *O*:*O*′] using the direct synthesis method, which is based on oxidative dissolution of a powdered metal in the presence of an organic ligand (Kokozay *et al.*, 2018 ▸; Li *et al.*, 2021 ▸). 
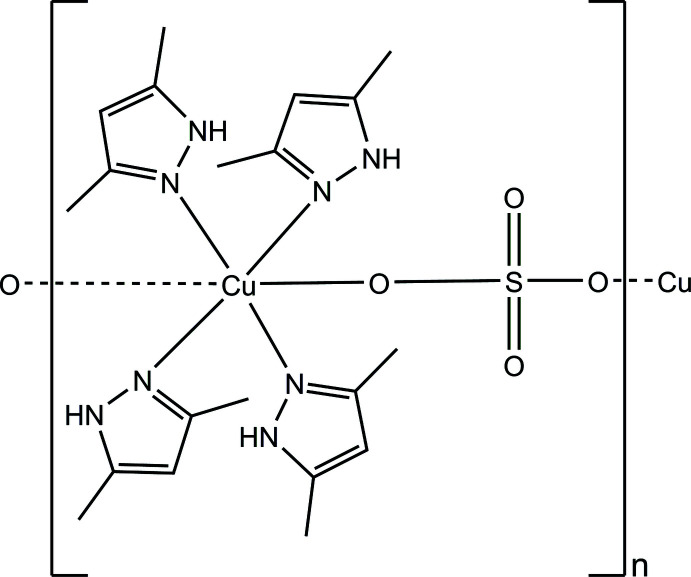


## Structural commentary

**Figure 1:**
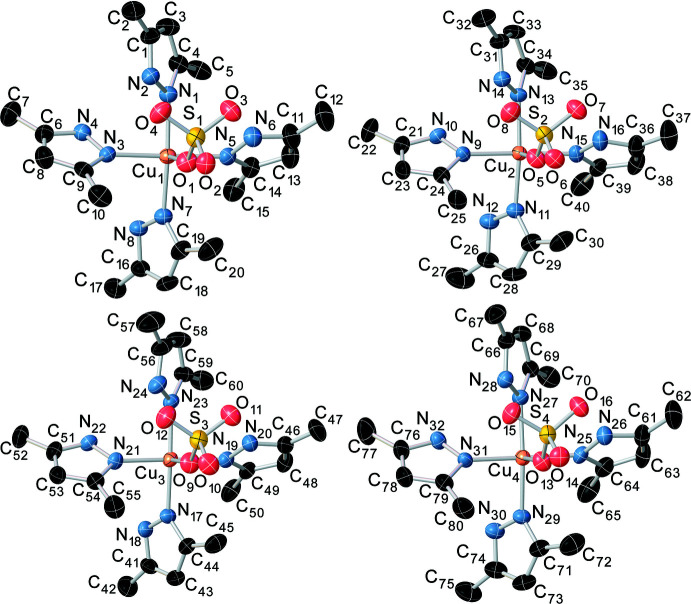
Representation of four [Cu(SO_4_)(Hdmpz)_4_] formula units in the structure of the title coordination polymer, with displacement ellipsoids at the 50% probability level.

**Figure 2:**
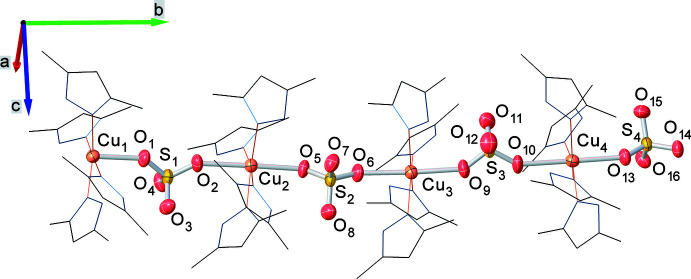
The asymmetric unit of the title compound. Selected pyrazole ring atoms are represented as wireframes. H atoms and hydrogen bonds are omitted for clarity.

**Figure 3:**
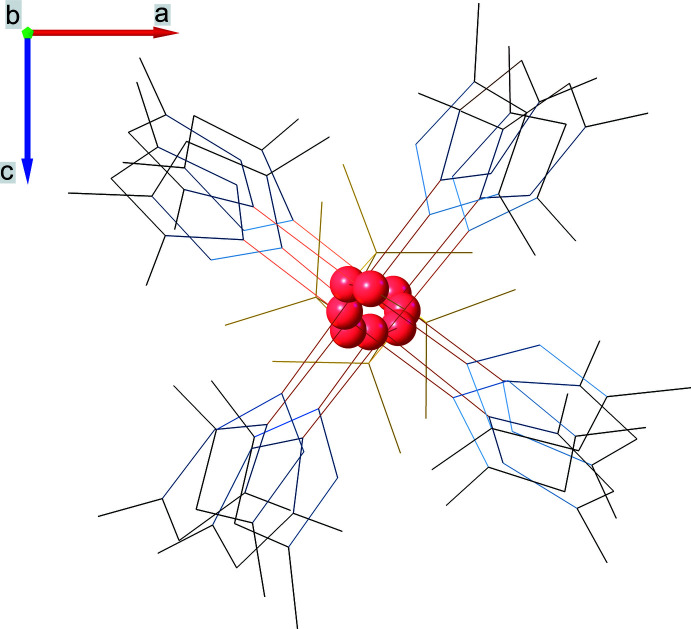
The spiral arrangement of the bridging oxygen atoms of the sulfate ligands, which bind [Cu(SO_4_)(Hdmpz)_4_] formula units along the *b*-axis direction. Bridging oxygen atoms of sulfate ligands are represented as red spheres, while all other atoms are depicted as wireframes. Hydrogen atoms are omitted for clarity.

The title coordination polymer crystallizes in the ortho­rhom­bic *Pna*2_1_ space group. The asymmetric unit is a chain consisting of four [Cu(Hdmpz)_4_SO_4_] formula units (Fig. 1 ▸) that are connected to each other by a μ_2_-sulfato-bridged ligand along the *b-*axis direction (Fig. 2 ▸). Each mononuclear unit [Cu(Hdmpz)_4_SO_4_] consists of four 3,5-dimethyl-1*H*-pyrazole mol­ecules, which are coordinated in a monodentate way, and one sulfate ligand that is connected by one oxygen atom to the copper ion. The octa­hedral coordination environment of each copper atom consists of four pyridine-like nitro­gen atoms of Hdmpz ligands, which occupy the equatorial positions, and two oxygen atoms of two SO_4_ ligands, which are in axial positions. The difference in lengths of the axial Cu—O and equatorial Cu—N bonds is at least 0.235 Å. Bond lengths between the central atom and the nitro­gen atoms in the equatorial position are approximately the same [in the range 2.028 (6) to 2.054 (6) Å]. The N1, N3, N5 and N7 nitro­gen atoms slightly deviate from of the equatorial plane [by −0.088 (3) Å for N1, 0.069 (3) Å for N3, 0.067 (3) Å for N5 and −0.086 (3) Å for N7]. The Cu1 atom is out of the equatorial plane, formed by four nitro­gen atoms, by 0.038 (3) Å. The N—Cu—N angles are practically right angles, in the range of 88.0 (2)–91.2 (2)°. The inter­metallic Cu⋯Cu distances between two neighboring [Cu(Hdmpz)_4_SO_4_] fragments within one asymmetric unit are in the range 7.0842 (12)–7.1554 (12) Å while the inter­chalcogenic S⋯S distances are in the range 7.166 (2)–7.223 (2) Å. Bridging oxygen atoms of sulfate ligands, which bind [Cu(Hdmpz)_4_SO_4_] formula units, are arranged in a spiral along the *b* axis (Fig. 3 ▸).

**Figure 4:**
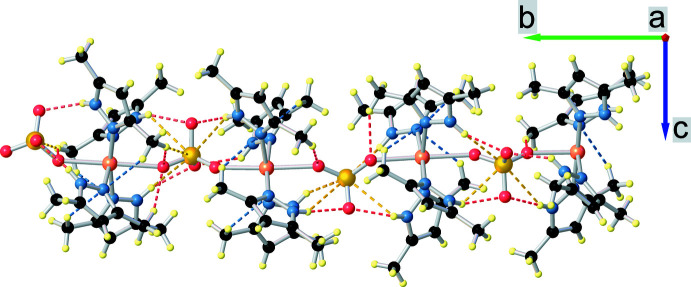
Intra­molecular hydrogen-bond network in the asymmetric unit of the title compound. Hydrogen bonds with the participation of oxygen atoms are indicated in red, blue for nitro­gen atoms and yellow for sulfur atoms. Hydrogen donors are carbon atoms of methyl groups and nitro­gen atoms of NH groups, while hydrogen acceptors are sulfur and oxygen atoms, and pyridine-like nitro­gen atoms of the pyrazole ring.

The mol­ecular structure of the complex is stabilized by weak intra­molecular hydrogen bonds in which hydrogen donors are carbon atoms (–CH_3_ groups at the 3 and 5 positions of the pyrazole ring) and pyrrole-like nitro­gen atoms of NH groups, while hydrogen acceptors are pyridine-like nitro­gen atoms of the neighboring pyrazole ligands and O and S atoms of the sulfate ligands. Significant contributions to the hydrogen-bond network are made by N—H⋯O hydrogen bonds with lengths in the range of 2.022 (5) to 2.437 (4) Å. Selected intra­molecular geometric parameters of hydrogen bonds are given in Table 1 ▸. The hydrogen-bond network in the asymmetric unit of the title compound is shown in Fig. 4 ▸. The torsion angle Cu1—Cu2—Cu3—Cu4 is −80.2 (2)° and S1—S2—S3–S4 is −97.8 (2)° and O1—O2— O5—O6, O5—O6—O9—O10 and O9—O10—O13—O14 are 36 (4), 25 (7) and 51 (3)°, respectively.

All pyrazole rings are oriented unsymmetrically in the mononuclear fragment. Thus, the planes of pyrazole rings N1/N2/C1/C3/C4 (pyrazole ligand near the Cu1 atom) and N9/N10/C21/C23/C24 (pyrazole ligand near the Cu2 atom) are oriented almost parallel to each other with a small deviation [plane normal to plane normal angle = 12.8 (3)°]. The plane-to-plane twist angle is 4.2 (4)°, the plane-to-plane fold angle is 13.4 (4)° and the plane-to-plane shift = 4.879 (18) Å. Within one [Cu(Hdmpz)_4_SO_4_] unit, pairs of pyrazole ring planes, for example N1/N2/C1/C3/C4, N7/N8/C16/C18/C19 and N3/N4/C6/C8/C9, N5/N6/C11/C13/C14, are placed in a non-parallel manner. The torsion angles N2—N1—N7—N8 and N4—N3—N5—N6 are 109.0 (6) and 111.3 (6)°, respectively.

## Supra­molecular features

**Figure 5:**
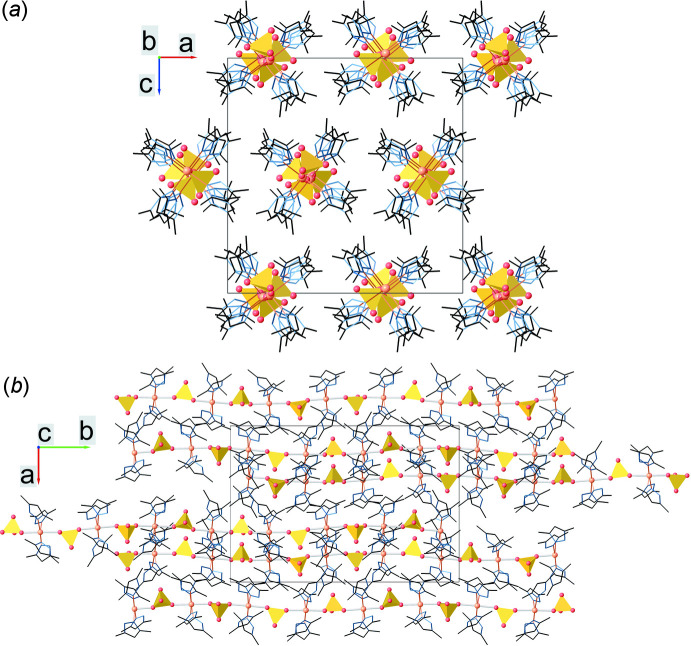
Crystal packing of the title compound viewed along the (*a*) *b*- and (*b*) *c*-axis directions: sulfate ligands are in a polyhedral representation with red spherical oxygen atoms, copper atoms are represented as orange spheres, while pyrazole rings atoms are depicted as wireframes. Hydrogen atoms are omitted for clarity.

The crystal structure (Fig. 5 ▸) is built up from polymeric chains packed parallel along the *b-*axis direction. The unit-cell dimensions can be explained because of the presence of four complex moieties in the asymmetric unit (*Z*′ = 4, *Z* = 16). As a result of the sulfate ligand rotation, there is a pairwise alternation of the terminal oxygen atoms (which are not involved in coordinating the copper atom) of the SO_4_ tetra­hedra. Within one chain the inter­metallic distance between two copper atoms, which are located at the edges of two neighboring asymmetric units, is 7.1625 (12) Å, while the inter­chalcogenic distance between the nearest sulfur atoms is 7.227 (2) Å. Polymeric chains, which are formed with the participation of bridging sulfate ligands, are stabilized by an extensive hydrogen-bond network. Neighboring chains are connected to each other by weak C—H⋯N and C—H⋯O hydrogen bonds. Geometric parameters for inter­molecular hydrogen bonds are given in Table 2 ▸.

## Hirshfeld surface analysis

**Figure 6:**
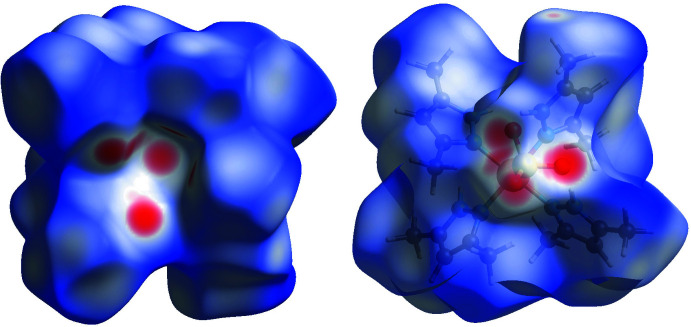
Two projections of Hirshfeld surfaces mapped over *d*_norm_ showing the inter­molecular inter­actions.

**Figure 7:**
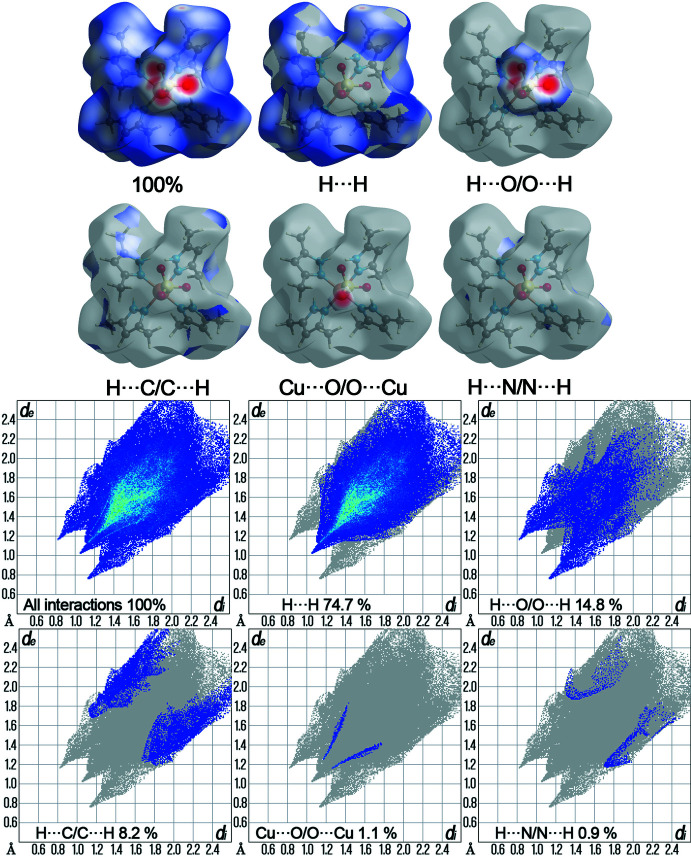
The overall two-dimensional fingerprint plot and those delineated into specified inter­actions. Hirshfeld surface representations with the function *d*_norm_ plotted onto the surface for the different inter­actions.

The Hirshfeld surface analysis was performed and the associated two-dimensional fingerprint plots generated using *Crystal Explorer 17.5* software (Spackman *et al.*, 2021 ▸), with a standard resolution of the three-dimensional *d*_norm_ surfaces plotted over a fixed color scale of −0.5511 (red) to 1.8416 (blue) a.u. The red spots in Fig. 6 ▸. represent short contacts and negative *d*_norm_ values on the surface corresponding to the inter­actions described above. The Hirshfeld surfaces mapped over *d*_norm_ are shown for the H⋯H, H⋯O/O⋯H, H⋯C/C⋯H, Cu⋯O/O⋯Cu and H⋯N/N⋯H contacts, the overall two-dimensional fingerprint plot and the decomposed two-dimensional fingerprint plots are given in Fig. 7 ▸. For the title coordination polymer, the most significant contributions to the overall crystal packing are from H⋯H (74.7%), H⋯O/O⋯H (14.8%) and H⋯C/C⋯H (8.2%) contacts. Small contributions of weak Cu⋯O/O⋯Cu (1.1%), H⋯N/N⋯H (0.9%) and N⋯O/O⋯N (0.2%) contacts have a negligible effect on the packing. The total contribution of contacts involving hydrogen atoms is 85.9%, for O atoms is 8.4%, C atoms 4.4%, N atoms 0.7% and Cu atoms 0.5%. These values were calculated using the *Crystal Explorer 17.5* software (Spackman *et al.*, 2021 ▸). A special filter ‘by elements’ was chosen during the calculation of the contributions of selected individual interactions to the total Hirshfeld surface. Qu­anti­tative physical properties of Hirshfeld surface for the title compound were also obtained, such as the mol­ecular volume (650.80 Å^3^), surface area (512.94 Å^2^), globularity (0.708), as well as sphericity (0.034). These properties provide significant information on the shape of the mol­ecules and may serve in the future to identify and establish correlations with other properties.

## Database survey

A search of the Cambridge Structural Database (CSD version 5.42, update February 2021; Groom *et al.*, 2016 ▸) for the Cu_2_(μ_2_-SO_4_)(Hpz)_4_ moiety [two Cu(Hpz)_2_ fragments connected through a bidentate-bridged SO_4_ ligand] revealed two hits: QITCAZ, a coordination compound based on 4-iodo-1*H*-pyrazole (Song *et al.*, 2013 ▸) and XACTUR, a 1*H*-pyrazole-containing complex (Shen *et al.*, 2004 ▸). These structures are similar to the title compound. Moreover there are 23 hits for the Cu(C_3_N_2_)_2_SO_4_ moiety, where C_3_N_2_ is the backbone of the pyrazole ring. Most similar to the title compound are two *catena*-[(μ_2_-sulfato)­bis­(3,5-dimethyl-1*H*-pyrazole)­aqua­copper(II)dihydrate] complexes: EHOMEU (Wang *et al.*, 2010 ▸) and EHOMEU01 (Gogoi *et al.*, 2019 ▸); FITCUI, a complex based on 2-thienyl-1*H*-pyrazole (Pettinari *et al.*, 2014 ▸); ZZZALD01 a tetra­kis­(pyrazole)(sulfato-*O*)copper(II) monohydrate (Shen *et al.*, 2004 ▸); two monohydrated tetra­pyrazole sulfato copper(II) complexes: LUNDAB (Kumar *et al.*, 2014 ▸) and LUNDAB01 (Zerguini *et al.*, 2019 ▸).

## Synthesis and crystallization

The synthesis of [Cu(SO_4_)(Hdmpz)_4_]_*n*_ was conducted at room temperature by the oxidative dissolution method as a result of the addition of a copper powder (1.56 mmol, 0.1 g) and anhydrous copper(II) sulfate (3.1 mmol, 0.5 g) mixture to an aceto­nitrile (9 ml) solution of 3,5-dimethyl-1*H*-pyrazole (4.68 mmol, 0.45g). The mixture was stirred without heating for three h with free air access until dissolution of the copper powder and a gray–blue precipitate of the product was obtained (the precipitate weight was 0.86 g). The precipitate was filtered off and the obtained green–blue solution was analyzed. Clear, intense blue crystals of the title compound suitable for X-ray analysis were obtained by slow evaporation of the solvent at room temperature in an open vessel. The relative yield of the single-crystal portion of the product with respect to the ligand was approximately 7%. The obtained blue crystals were studied by elemental analysis (calculated for C_20_H_32_CuN_8_O_4_S: C 44.1%, H 5.9%, N 20.6%, found: C 44.5%, H 6.3%, N 21%). The elemental analysis data of the obtained grey–blue precipitate was: found C 36.8%, H 5.5%, N 17.2%. IR spectra of the starting 3,5-dimethyl-1*H*-pyrazole, grey–blue precipitate and clear, intense blue crystals of the title coordination polymer are given in the supporting information for this article.

## Refinement

Crystal data, data collection and structure refinement details are summarized in Table 3 ▸. Refinement of the N—H bond lengths was attempted, but this provided unrealistic values. Thus, hydrogens were placed at calculated positions and refined as riding with *U*_iso_(H) = 1.2*U*_eq_(N, C) or 1.5*U*_eq_(C-meth­yl). The crystal studied was refined as a two-component inversion twin.

## Supplementary Material

Crystal structure: contains datablock(s) I. DOI: 10.1107/S2056989022002894/dj2038sup1.cif

Structure factors: contains datablock(s) I. DOI: 10.1107/S2056989022002894/dj2038Isup2.hkl

Supporting information file. DOI: 10.1107/S2056989022002894/dj2038Isup3.cdx

IR spectrum of the starting 3,5-dimethyl-1H-pyrazole. DOI: 10.1107/S2056989022002894/dj2038sup4.txt

IR spectrum of the grey-blue precipitate. DOI: 10.1107/S2056989022002894/dj2038sup5.txt

IR spectrum of intense blue crystals of the title coordination polymer. DOI: 10.1107/S2056989022002894/dj2038sup6.txt

CCDC reference: 2158601

Additional supporting information:  crystallographic information; 3D view; checkCIF report

## Figures and Tables

**Table 1:** Geometry of intramolecular hydrogen bonds (Å, °)

*D*—H⋯*A*	*D*—H	H⋯*A*	*D*⋯*A*	*D*—H⋯*A*
N2—H2⋯O4	0.86	2.08	2.792 (7)	139
N6—H6⋯O3	0.86	2.04	2.889 (7)	168
N10—H10⋯O3	0.86	2.11	2.869 (7)	146
N12—H12⋯O4	0.86	2.12	2.951 (8)	163
N14—H14⋯O8	0.86	2.10	2.835 (7)	143
N16—H16⋯O5	0.86	2.44	2.889 (7)	114
N16—H16⋯O7	0.86	2.04	2.894 (8)	173
N18—H18⋯O6	0.86	2.39	2.866 (8)	116
N18—H18⋯O8	0.86	2.14	2.988 (7)	169
N20—H20⋯O9	0.86	2.41	2.885 (9)	115
N20—H20⋯O11	0.86	2.08	2.933 (7)	171
N22—H22⋯O7	0.86	2.05	2.828 (7)	150
N24—H24⋯O12	0.86	2.16	2.840 (8)	135
N26—H26⋯O16	0.86	2.02	2.875 (7)	171
N28—H28⋯O15	0.86	2.07	2.803 (7)	143
N30—H30⋯O10	0.86	2.31	2.817 (8)	118
N30—H30⋯O12	0.86	2.24	3.083 (8)	165
N32—H32⋯O11	0.86	2.12	2.857 (7)	144
C30—H30*C*⋯O5	0.96	2.39	3.213 (11)	144
C50—H50*A*⋯O6	0.96	2.23	3.124 (9)	155
C65—H65*B*⋯O10	0.96	2.27	3.192 (11)	160
C70—H70*B*⋯O10	0.96	2.35	3.116 (10)	137

**Table 2:** Geometric parameters of inter­molecular hydrogen bonds (Å, °)

C2—H2*A*⋯N16^i^	0.96	3.01	3.722 (10)	132
C2—H2*A*⋯O7^i^	0.96	28	3.806 (9)	146
C53—H53⋯N8^ii^	0.93	3.07	3.66 (1)	123
C32—H32*B*⋯N32^iii^	0.96	3.00	3.792 (10)	140
C32—H32*B*⋯N31^iii^	0.96	3.17	3.984 (10)	143
C32—H32*B*⋯N28^iii^	0.96	2.87	3.735 (11)	150

Symmetry codes: (i) 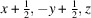 ; (ii) 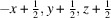 ; (iii) 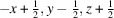 .

**Table 3:** Experimental details

Crystal data	
Chemical formula	[Cu(SO_4_)(C_5_H_8_N_2_)_4_]
*M*_r_	544.13
Crystal system, space group	Orthorhombic, *P* *n* *a*2_1_
Temperature (K)	293
*a*, *b*, *c* (Å)	19.3656 (6), 28.4032 (6), 19.3456 (5)
*V* (Å^3^)	10641.0 (5)
*Z*	16
Radiation type	Mo *K*α
μ (mm^−1^)	0.94
Crystal size (mm)	0.35 × 0.25 × 0.25

Data collection	
Diffractometer	Rigaku Oxford Diffraction Xcalibur, Eos
Absorption correction	Multi-scan (*CrysAlis PRO*; Rigaku OD, 2021 ▸)
*T*_min_, *T*_max_	0.907, 1.000
No. of measured, independent and observed [*I* > 2σ(*I*)] reflections	119732, 22862, 14389
*R*_int_	0.062
(sin θ/λ)_max_ (Å^−1^)	0.690

Refinement	
*R*[*F* ^2^ > 2σ(*F* ^2^)], *wR*(*F* ^2^), *S*	0.054, 0.133, 1.03
No. of reflections	22862
No. of parameters	1234
No. of restraints	1
H-atom treatment	H-atom parameters constrained
Δρ_max_, Δρ_min_ (e Å^−3^)	1.19, −0.30
Absolute structure	Refined as an inversion twin
Absolute structure parameter	0.479 (15)

Computer programs: *CrysAlis PRO* (Rigaku OD, 2021 ▸), *SHELXT2018/2* (Sheldrick, 2015*a* ▸), *SHELXL2018/3* (Sheldrick, 2015*b* ▸) and *OLEX2* (Dolomanov *et al.*, 2009 ▸).

## supplementary crystallographic information

### Crystal data

**Table:**

[Cu(SO_4_)(C_5_H_8_N_2_)_4_]	*D*_x_ = 1.359 Mg m^−^^3^
*M**_r_* = 544.13	Mo *K*α radiation, λ = 0.71073 Å
Orthorhombic, *P**n**a*2_1_	Cell parameters from 24752 reflections
*a* = 19.3656 (6) Å	θ = 2.1–24.0°
*b* = 28.4032 (6) Å	µ = 0.94 mm^−^^1^
*c* = 19.3456 (5) Å	*T* = 293 K
*V* = 10641.0 (5) Å^3^	Prism, clear intense blue
*Z* = 16	0.35 × 0.25 × 0.25 mm
*F*(000) = 4560	

### Data collection

**Table:**

Rigaku Oxford Diffraction Xcalibur, Eos diffractometer	22862 independent reflections
Radiation source: fine-focus sealed X-ray tube, Enhance (Mo) X-ray Source	14389 reflections with *I* > 2σ(*I*)
Graphite monochromator	*R*_int_ = 0.062
Detector resolution: 16.1593 pixels mm^-1^	θ_max_ = 29.4°, θ_min_ = 1.7°
ω scans	*h* = −26→26
Absorption correction: multi-scan (*CrysAlis PRO*; Rigaku OD, 2021)	*k* = −39→36
*T*_min_ = 0.907, *T*_max_ = 1.000	*l* = −22→26
119732 measured reflections	

### Refinement

**Table:**

Refinement on *F*^2^	Hydrogen site location: mixed
Least-squares matrix: full	H-atom parameters constrained
*R*[*F*^2^ > 2σ(*F*^2^)] = 0.054	*w* = 1/[σ^2^(*F*_o_^2^) + (0.0546*P*)^2^ + 4.6894*P*] where *P* = (*F*_o_^2^ + 2*F*_c_^2^)/3
*wR*(*F*^2^) = 0.133	(Δ/σ)_max_ = 0.001
*S* = 1.03	Δρ_max_ = 1.19 e Å^−^^3^
22862 reflections	Δρ_min_ = −0.30 e Å^−^^3^
1234 parameters	Absolute structure: Refined as an inversion twin
1 restraint	Absolute structure parameter: 0.479 (15)
Primary atom site location: dual	

### Special details

**Table:**

Geometry. All esds (except the esd in the dihedral angle between two l.s. planes) are estimated using the full covariance matrix. The cell esds are taken into account individually in the estimation of esds in distances, angles and torsion angles; correlations between esds in cell parameters are only used when they are defined by crystal symmetry. An approximate (isotropic) treatment of cell esds is used for estimating esds involving l.s. planes.
Refinement. Refined as a 2-component inversion twin.

### Fractional atomic coordinates and isotropic or equivalent isotropic displacement parameters (Å^2^)

**Table:**

	*x*	*y*	*z*	*U*_iso_*/*U*_eq_	
Cu1	0.32732 (4)	0.08172 (3)	0.47991 (5)	0.0381 (2)	
Cu2	0.35680 (4)	0.33019 (3)	0.49183 (4)	0.0366 (2)	
Cu3	0.34772 (4)	0.57922 (3)	0.51712 (5)	0.0386 (2)	
Cu4	0.31888 (4)	0.83027 (3)	0.50659 (4)	0.0383 (2)	
S1	0.38041 (9)	0.20005 (5)	0.50836 (11)	0.0453 (4)	
S2	0.33477 (9)	0.45147 (5)	0.54082 (11)	0.0425 (5)	
S3	0.29310 (9)	0.69962 (5)	0.48845 (12)	0.0482 (5)	
S4	0.34086 (9)	0.94986 (5)	0.45316 (11)	0.0415 (4)	
O1	0.3364 (2)	0.16205 (14)	0.4818 (3)	0.0518 (14)	
O2	0.3542 (2)	0.24597 (13)	0.4860 (3)	0.0507 (13)	
O3	0.3800 (3)	0.19749 (17)	0.5846 (3)	0.0574 (15)	
O4	0.4509 (2)	0.19497 (15)	0.4824 (3)	0.0612 (15)	
O5	0.3613 (2)	0.41241 (13)	0.4991 (3)	0.0495 (14)	
O6	0.3581 (2)	0.49625 (14)	0.5126 (3)	0.0526 (14)	
O7	0.2586 (2)	0.44992 (15)	0.5392 (3)	0.0572 (14)	
O8	0.3598 (3)	0.44762 (16)	0.6120 (3)	0.0603 (16)	
O9	0.3357 (2)	0.66123 (14)	0.5160 (4)	0.0570 (15)	
O10	0.3188 (3)	0.74469 (14)	0.5145 (3)	0.0603 (15)	
O11	0.2977 (3)	0.69918 (17)	0.4129 (3)	0.0629 (16)	
O12	0.2213 (3)	0.69417 (17)	0.5107 (4)	0.0710 (16)	
O13	0.3157 (2)	0.91247 (13)	0.4997 (3)	0.0486 (14)	
O14	0.3186 (2)	0.99611 (14)	0.4783 (3)	0.0516 (14)	
O15	0.3144 (3)	0.94324 (18)	0.3832 (3)	0.0599 (16)	
O16	0.4168 (2)	0.94793 (16)	0.4532 (3)	0.0537 (14)	
N1	0.4125 (3)	0.07577 (18)	0.5413 (3)	0.0406 (14)	
N2	0.4699 (3)	0.10224 (17)	0.5263 (3)	0.0421 (13)	
H2	0.471469	0.123444	0.494624	0.050*	
N3	0.3918 (3)	0.07707 (17)	0.3958 (3)	0.0371 (13)	
N4	0.4314 (3)	0.03765 (18)	0.3906 (3)	0.0406 (14)	
H4	0.431493	0.015166	0.420404	0.049*	
N5	0.2660 (3)	0.08838 (18)	0.5646 (3)	0.0402 (14)	
N6	0.2834 (3)	0.12205 (18)	0.6108 (3)	0.0492 (15)	
H6	0.316691	0.141689	0.604779	0.059*	
N7	0.2437 (3)	0.07873 (18)	0.4170 (3)	0.0404 (15)	
N8	0.2337 (3)	0.03829 (18)	0.3801 (3)	0.0404 (14)	
H8	0.261953	0.014945	0.380052	0.048*	
N9	0.4407 (3)	0.32578 (17)	0.5546 (3)	0.0363 (13)	
N10	0.4483 (3)	0.28685 (18)	0.5947 (3)	0.0412 (14)	
H10	0.415730	0.266851	0.601978	0.049*	
N11	0.4228 (3)	0.32439 (18)	0.4100 (3)	0.0401 (14)	
N12	0.4621 (3)	0.28530 (19)	0.4079 (3)	0.0473 (15)	
H12	0.459640	0.262546	0.437135	0.057*	
N13	0.2952 (3)	0.32716 (17)	0.5767 (3)	0.0374 (14)	
N14	0.3102 (3)	0.35464 (17)	0.6314 (3)	0.0401 (14)	
H14	0.342157	0.375686	0.630656	0.048*	
N15	0.2751 (3)	0.33851 (18)	0.4275 (3)	0.0410 (15)	
N16	0.2360 (3)	0.37800 (19)	0.4357 (3)	0.0507 (16)	
H16	0.241034	0.397862	0.468889	0.061*	
N17	0.4299 (3)	0.57622 (18)	0.5830 (3)	0.0408 (14)	
N18	0.4410 (3)	0.53691 (18)	0.6198 (3)	0.0425 (14)	
H18	0.413143	0.513345	0.620048	0.051*	
N19	0.4139 (3)	0.58823 (18)	0.4359 (3)	0.0400 (14)	
N20	0.4009 (3)	0.62485 (18)	0.3930 (3)	0.0431 (14)	
H20	0.367047	0.644127	0.398058	0.052*	
N21	0.2827 (3)	0.57547 (17)	0.5999 (3)	0.0407 (14)	
N22	0.2421 (3)	0.53702 (18)	0.6081 (3)	0.0416 (15)	
H22	0.236905	0.515774	0.576872	0.050*	
N23	0.2662 (3)	0.57376 (18)	0.4494 (3)	0.0414 (14)	
N24	0.2100 (3)	0.60092 (19)	0.4590 (4)	0.0542 (16)	
H24	0.204744	0.619756	0.493354	0.065*	
N25	0.4015 (3)	0.84053 (18)	0.5680 (3)	0.0398 (14)	
N26	0.4448 (3)	0.87635 (17)	0.5545 (3)	0.0435 (14)	
H26	0.438945	0.895919	0.521062	0.052*	
N27	0.3831 (3)	0.82429 (18)	0.4231 (3)	0.0389 (14)	
N28	0.3720 (3)	0.85335 (18)	0.3675 (3)	0.0409 (14)	
H28	0.338936	0.873473	0.365124	0.049*	
N29	0.2546 (3)	0.82565 (18)	0.5892 (3)	0.0409 (14)	
N30	0.2132 (3)	0.78693 (19)	0.5931 (3)	0.0476 (15)	
H30	0.213789	0.764259	0.563671	0.057*	
N31	0.2358 (3)	0.82528 (17)	0.4430 (3)	0.0362 (13)	
N32	0.2305 (3)	0.78826 (18)	0.3996 (3)	0.0433 (15)	
H32	0.263325	0.768555	0.392030	0.052*	
C1	0.5230 (4)	0.0909 (3)	0.5672 (4)	0.055 (2)	
C2	0.5895 (4)	0.1169 (3)	0.5600 (5)	0.076 (3)	
H2A	0.621893	0.097751	0.535050	0.114*	
H2B	0.581603	0.145661	0.535100	0.114*	
H2C	0.607663	0.123991	0.604930	0.114*	
C3	0.5007 (4)	0.0559 (3)	0.6100 (5)	0.064 (2)	
H3	0.526299	0.040852	0.644164	0.077*	
C4	0.4319 (4)	0.0468 (2)	0.5924 (4)	0.0472 (19)	
C5	0.3846 (4)	0.0124 (3)	0.6251 (5)	0.067 (2)	
H5A	0.356433	0.028130	0.658716	0.100*	
H5B	0.355543	−0.001530	0.590356	0.100*	
H5C	0.411053	−0.011900	0.647336	0.100*	
C6	0.4698 (4)	0.0382 (2)	0.3339 (4)	0.0493 (19)	
C7	0.5150 (4)	−0.0028 (3)	0.3150 (5)	0.074 (3)	
H7A	0.541754	−0.012111	0.354541	0.110*	
H7B	0.486784	−0.028761	0.300331	0.110*	
H7C	0.545514	0.006079	0.278111	0.110*	
C8	0.4558 (4)	0.0797 (2)	0.3010 (4)	0.057 (2)	
H8A	0.475469	0.090205	0.259971	0.069*	
C9	0.4068 (4)	0.1032 (2)	0.3403 (4)	0.0433 (17)	
C10	0.3741 (5)	0.1494 (2)	0.3269 (4)	0.064 (2)	
H10A	0.370788	0.154240	0.277892	0.095*	
H10B	0.328688	0.150070	0.346832	0.095*	
H10C	0.401648	0.173990	0.346972	0.095*	
C11	0.2429 (5)	0.1213 (3)	0.6671 (5)	0.064 (2)	
C12	0.2525 (5)	0.1557 (3)	0.7255 (5)	0.091 (3)	
H12A	0.228809	0.144454	0.765843	0.137*	
H12B	0.300869	0.158834	0.735583	0.137*	
H12C	0.234159	0.185804	0.712433	0.137*	
C13	0.1985 (5)	0.0846 (3)	0.6565 (5)	0.065 (2)	
H13	0.163781	0.074778	0.686407	0.078*	
C14	0.2144 (4)	0.0648 (2)	0.5933 (4)	0.0498 (19)	
C15	0.1806 (4)	0.0235 (2)	0.5586 (5)	0.065 (2)	
H15A	0.164851	0.032739	0.513512	0.098*	
H15B	0.213211	−0.001791	0.554232	0.098*	
H15C	0.141941	0.013189	0.585722	0.098*	
C16	0.1745 (4)	0.0393 (2)	0.3441 (4)	0.0472 (18)	
C17	0.1537 (4)	−0.0010 (3)	0.2990 (6)	0.069 (3)	
H17A	0.183502	−0.002395	0.259473	0.104*	
H17B	0.106820	0.003300	0.284058	0.104*	
H17C	0.157235	−0.029876	0.324644	0.104*	
C18	0.1448 (4)	0.0824 (3)	0.3581 (5)	0.058 (2)	
H18A	0.103391	0.093839	0.340651	0.069*	
C19	0.1889 (4)	0.1050 (2)	0.4032 (4)	0.0492 (19)	
C20	0.1813 (4)	0.1533 (2)	0.4351 (5)	0.074 (3)	
H20A	0.150287	0.151529	0.473782	0.111*	
H20B	0.225577	0.164219	0.450522	0.111*	
H20C	0.163077	0.174749	0.401402	0.111*	
C21	0.5111 (4)	0.2829 (2)	0.6214 (4)	0.0485 (18)	
C22	0.5304 (5)	0.2404 (3)	0.6634 (4)	0.069 (2)	
H22A	0.559190	0.249827	0.701352	0.104*	
H22B	0.554996	0.218450	0.634818	0.104*	
H22C	0.489284	0.225722	0.680924	0.104*	
C23	0.5467 (4)	0.3217 (3)	0.6007 (4)	0.0514 (19)	
H23	0.591877	0.329426	0.612165	0.062*	
C24	0.5018 (4)	0.3476 (2)	0.5585 (4)	0.0426 (16)	
C25	0.5174 (4)	0.3928 (2)	0.5231 (5)	0.060 (2)	
H25A	0.566328	0.398168	0.523457	0.090*	
H25B	0.494508	0.418130	0.546674	0.090*	
H25C	0.501375	0.391373	0.476133	0.090*	
C26	0.5061 (4)	0.2867 (3)	0.3537 (4)	0.056 (2)	
C27	0.5556 (6)	0.2466 (3)	0.3427 (6)	0.104 (4)	
H27A	0.588553	0.254903	0.307665	0.156*	
H27B	0.579403	0.239943	0.385145	0.156*	
H27C	0.530273	0.219273	0.328355	0.156*	
C28	0.4935 (5)	0.3277 (3)	0.3192 (4)	0.064 (2)	
H28A	0.515504	0.338284	0.279364	0.077*	
C29	0.4408 (4)	0.3507 (2)	0.3557 (4)	0.0505 (19)	
C30	0.4076 (5)	0.3971 (3)	0.3412 (4)	0.071 (3)	
H30A	0.362296	0.392072	0.322493	0.107*	
H30B	0.435085	0.414249	0.308444	0.107*	
H30C	0.404123	0.414809	0.383313	0.107*	
C31	0.2715 (4)	0.3463 (2)	0.6863 (4)	0.0489 (18)	
C32	0.2785 (5)	0.3752 (3)	0.7511 (4)	0.071 (2)	
H32A	0.260913	0.406319	0.742753	0.106*	
H32B	0.252743	0.360759	0.787763	0.106*	
H32C	0.326293	0.377149	0.763903	0.106*	
C33	0.2287 (4)	0.3101 (3)	0.6676 (4)	0.059 (2)	
H33	0.195432	0.295838	0.695224	0.070*	
C34	0.2444 (4)	0.2987 (2)	0.5991 (4)	0.0462 (19)	
C35	0.2102 (4)	0.2627 (3)	0.5545 (5)	0.067 (2)	
H35A	0.227412	0.232031	0.566120	0.101*	
H35B	0.161182	0.263641	0.562070	0.101*	
H35C	0.219822	0.269301	0.506840	0.101*	
C36	0.1892 (4)	0.3819 (3)	0.3860 (5)	0.062 (2)	
C37	0.1406 (5)	0.4231 (3)	0.3818 (6)	0.099 (4)	
H37A	0.093811	0.411965	0.382856	0.148*	
H37B	0.148371	0.443765	0.420305	0.148*	
H37C	0.148481	0.439946	0.339455	0.148*	
C38	0.1967 (5)	0.3428 (3)	0.3447 (5)	0.065 (2)	
H38	0.170690	0.335538	0.305757	0.078*	
C39	0.2504 (4)	0.3164 (2)	0.3720 (4)	0.0490 (19)	
C40	0.2795 (5)	0.2713 (3)	0.3473 (5)	0.073 (3)	
H40A	0.256185	0.261897	0.305781	0.110*	
H40B	0.327845	0.275217	0.338011	0.110*	
H40C	0.273345	0.247627	0.382141	0.110*	
C41	0.4994 (4)	0.5381 (3)	0.6556 (4)	0.0498 (19)	
C42	0.5231 (4)	0.4972 (3)	0.6985 (5)	0.077 (3)	
H42A	0.516980	0.504359	0.746621	0.116*	
H42B	0.571074	0.491173	0.689528	0.116*	
H42C	0.496494	0.469786	0.686875	0.116*	
C43	0.5285 (4)	0.5809 (3)	0.6422 (4)	0.056 (2)	
H43	0.569799	0.592359	0.659965	0.067*	
C44	0.4843 (4)	0.6040 (2)	0.5965 (4)	0.0471 (18)	
C45	0.4919 (4)	0.6522 (2)	0.5650 (5)	0.070 (2)	
H45A	0.539299	0.661929	0.568093	0.104*	
H45B	0.478179	0.651109	0.517373	0.104*	
H45C	0.463279	0.674139	0.589483	0.104*	
C46	0.4464 (4)	0.6273 (3)	0.3424 (4)	0.0526 (19)	
C47	0.4456 (5)	0.6662 (3)	0.2898 (4)	0.078 (3)	
H47A	0.452922	0.653232	0.244593	0.117*	
H47B	0.401622	0.681892	0.291063	0.117*	
H47C	0.481512	0.688432	0.300053	0.117*	
C48	0.4908 (4)	0.5902 (3)	0.3515 (4)	0.056 (2)	
H48	0.528511	0.582495	0.324045	0.068*	
C49	0.4680 (4)	0.5665 (2)	0.4099 (4)	0.0431 (18)	
C50	0.4983 (4)	0.5240 (2)	0.4440 (5)	0.066 (2)	
H50A	0.462492	0.506713	0.466947	0.098*	
H50B	0.532356	0.533715	0.477141	0.098*	
H50C	0.519666	0.504384	0.409681	0.098*	
C51	0.2114 (4)	0.5354 (2)	0.6688 (4)	0.0495 (19)	
C52	0.1679 (4)	0.4945 (3)	0.6911 (6)	0.072 (3)	
H52A	0.183295	0.466551	0.667829	0.108*	
H52B	0.172145	0.490291	0.740129	0.108*	
H52C	0.120465	0.500471	0.679529	0.108*	
C53	0.2305 (4)	0.5758 (3)	0.7027 (4)	0.0517 (19)	
H53	0.216045	0.585499	0.746269	0.062*	
C54	0.2752 (4)	0.5990 (2)	0.6595 (4)	0.0454 (17)	
C55	0.3139 (5)	0.6447 (3)	0.6737 (5)	0.072 (3)	
H55A	0.293584	0.669771	0.647370	0.109*	
H55B	0.361446	0.641088	0.660726	0.109*	
H55C	0.311095	0.652069	0.722096	0.109*	
C56	0.1634 (4)	0.5951 (3)	0.4085 (5)	0.069 (3)	
C57	0.0974 (5)	0.6235 (3)	0.4060 (6)	0.105 (4)	
H57A	0.060225	0.605184	0.424750	0.157*	
H57B	0.103045	0.651724	0.432740	0.157*	
H57C	0.087055	0.631684	0.358930	0.157*	
C58	0.1910 (5)	0.5612 (3)	0.3649 (5)	0.078 (3)	
H58	0.170809	0.548858	0.325256	0.094*	
C59	0.2541 (4)	0.5495 (3)	0.3921 (5)	0.053 (2)	
C60	0.3040 (5)	0.5152 (3)	0.3616 (5)	0.076 (3)	
H60A	0.292885	0.510294	0.313798	0.114*	
H60B	0.350055	0.527494	0.365318	0.114*	
H60C	0.301045	0.485884	0.386008	0.114*	
C61	0.4981 (4)	0.8783 (3)	0.5986 (4)	0.059 (2)	
C62	0.5536 (5)	0.9155 (3)	0.5916 (6)	0.096 (4)	
H62A	0.549922	0.937439	0.629168	0.143*	
H62B	0.547886	0.931859	0.548573	0.143*	
H62C	0.598204	0.900764	0.592679	0.143*	
C63	0.4896 (4)	0.8416 (3)	0.6425 (5)	0.062 (2)	
H63	0.518563	0.833641	0.679017	0.075*	
C64	0.4295 (4)	0.8180 (2)	0.6230 (4)	0.050 (2)	
C65	0.3958 (5)	0.7755 (3)	0.6532 (5)	0.077 (3)	
H65A	0.366272	0.784778	0.690652	0.115*	
H65B	0.368892	0.760019	0.618272	0.115*	
H65C	0.430652	0.754378	0.670042	0.115*	
C66	0.4183 (4)	0.8468 (2)	0.3178 (4)	0.0473 (18)	
C67	0.4217 (5)	0.8754 (3)	0.2557 (4)	0.068 (2)	
H67A	0.468548	0.877074	0.239690	0.102*	
H67B	0.393268	0.861564	0.220500	0.102*	
H67C	0.405298	0.906574	0.265840	0.102*	
C68	0.4596 (4)	0.8108 (3)	0.3422 (4)	0.055 (2)	
H68	0.496530	0.797369	0.318549	0.066*	
C69	0.4375 (4)	0.7979 (2)	0.4067 (4)	0.0422 (19)	
C70	0.4662 (4)	0.7622 (3)	0.4553 (5)	0.067 (2)	
H70A	0.483103	0.777673	0.496045	0.100*	
H70B	0.430573	0.740303	0.467885	0.100*	
H70C	0.503403	0.745543	0.433365	0.100*	
C71	0.2370 (4)	0.8513 (2)	0.6433 (4)	0.0496 (19)	
C72	0.2728 (5)	0.8978 (3)	0.6572 (5)	0.080 (3)	
H72A	0.244398	0.923201	0.640899	0.120*	
H72B	0.280248	0.901271	0.706029	0.120*	
H72C	0.316388	0.898421	0.633629	0.120*	
C73	0.1851 (4)	0.8299 (3)	0.6812 (4)	0.063 (2)	
H73	0.163823	0.841381	0.720887	0.076*	
C74	0.1716 (4)	0.7884 (3)	0.6479 (5)	0.058 (2)	
C75	0.1211 (5)	0.7494 (3)	0.6620 (6)	0.086 (3)	
H75A	0.075626	0.762464	0.667102	0.129*	
H75B	0.121366	0.727504	0.624292	0.129*	
H75C	0.133946	0.733504	0.703852	0.129*	
C76	0.1681 (4)	0.7857 (2)	0.3697 (4)	0.052 (2)	
C77	0.1515 (5)	0.7477 (3)	0.3181 (6)	0.090 (3)	
H77A	0.158352	0.717433	0.339191	0.135*	
H77B	0.104222	0.750633	0.303761	0.135*	
H77C	0.181192	0.750673	0.278651	0.135*	
C78	0.1310 (4)	0.8229 (3)	0.3945 (4)	0.055 (2)	
H78	0.085662	0.830445	0.383381	0.066*	
C79	0.1740 (3)	0.8474 (2)	0.4397 (4)	0.0445 (17)	
C80	0.1588 (4)	0.8913 (2)	0.4792 (5)	0.068 (2)	
H80A	0.115620	0.904245	0.463907	0.102*	
H80B	0.155950	0.884095	0.527647	0.102*	
H80C	0.195000	0.913715	0.471527	0.102*	

### Atomic displacement parameters (Å^2^)

**Table:**

	*U*^11^	*U*^22^	*U*^33^	*U*^12^	*U*^13^	*U*^23^
Cu1	0.0354 (4)	0.0381 (4)	0.0408 (6)	−0.0023 (3)	−0.0030 (4)	−0.0003 (4)
Cu2	0.0354 (4)	0.0365 (4)	0.0380 (5)	−0.0001 (3)	0.0027 (4)	0.0004 (4)
Cu3	0.0340 (4)	0.0375 (4)	0.0443 (6)	−0.0002 (3)	0.0000 (4)	0.0033 (4)
Cu4	0.0361 (4)	0.0427 (4)	0.0363 (5)	−0.0003 (3)	−0.0013 (4)	0.0022 (4)
S1	0.0503 (10)	0.0279 (7)	0.0578 (13)	−0.0068 (7)	0.0034 (10)	−0.0005 (8)
S2	0.0462 (10)	0.0256 (8)	0.0556 (13)	−0.0035 (7)	−0.0002 (9)	−0.0013 (8)
S3	0.0541 (11)	0.0306 (8)	0.0598 (14)	0.0086 (7)	0.0045 (10)	−0.0009 (9)
S4	0.0467 (10)	0.0276 (8)	0.0502 (12)	0.0015 (7)	−0.0062 (9)	0.0039 (8)
O1	0.059 (3)	0.030 (2)	0.066 (4)	−0.011 (2)	−0.001 (3)	−0.001 (3)
O2	0.063 (3)	0.028 (2)	0.061 (4)	−0.0023 (19)	0.008 (3)	0.004 (3)
O3	0.072 (4)	0.052 (3)	0.049 (4)	−0.020 (3)	−0.003 (3)	0.001 (3)
O4	0.049 (3)	0.048 (3)	0.086 (4)	0.004 (2)	0.008 (3)	0.009 (3)
O5	0.050 (3)	0.030 (2)	0.069 (4)	0.0009 (18)	0.003 (3)	−0.012 (2)
O6	0.057 (3)	0.030 (2)	0.071 (4)	−0.0073 (19)	0.003 (3)	0.001 (3)
O7	0.045 (3)	0.048 (3)	0.078 (4)	−0.005 (2)	0.003 (3)	−0.014 (3)
O8	0.077 (4)	0.048 (3)	0.056 (4)	−0.010 (3)	−0.008 (3)	0.004 (3)
O9	0.070 (3)	0.029 (2)	0.072 (4)	0.008 (2)	0.002 (3)	0.004 (3)
O10	0.087 (4)	0.025 (2)	0.069 (4)	0.007 (2)	−0.001 (3)	−0.003 (3)
O11	0.076 (4)	0.057 (3)	0.055 (4)	0.020 (3)	0.003 (3)	−0.004 (3)
O12	0.057 (3)	0.062 (3)	0.094 (5)	0.002 (2)	0.016 (3)	−0.001 (3)
O13	0.051 (3)	0.029 (2)	0.066 (4)	−0.0021 (18)	−0.001 (3)	0.007 (2)
O14	0.061 (3)	0.029 (2)	0.064 (4)	0.005 (2)	0.000 (3)	0.001 (3)
O15	0.073 (4)	0.060 (3)	0.046 (4)	0.010 (3)	−0.017 (3)	−0.002 (3)
O16	0.043 (3)	0.050 (3)	0.069 (4)	−0.001 (2)	−0.003 (3)	0.017 (3)
N1	0.039 (3)	0.038 (3)	0.044 (4)	−0.005 (2)	−0.002 (3)	0.004 (3)
N2	0.040 (3)	0.037 (3)	0.049 (4)	−0.003 (2)	−0.002 (3)	−0.001 (3)
N3	0.035 (3)	0.032 (3)	0.044 (4)	0.000 (2)	−0.001 (3)	−0.002 (3)
N4	0.041 (3)	0.040 (3)	0.041 (4)	−0.007 (3)	−0.001 (3)	0.005 (3)
N5	0.041 (3)	0.032 (3)	0.047 (4)	0.001 (2)	0.001 (3)	−0.004 (3)
N6	0.055 (4)	0.039 (3)	0.054 (4)	−0.010 (3)	0.003 (3)	−0.007 (3)
N7	0.040 (3)	0.035 (3)	0.047 (4)	−0.001 (3)	−0.003 (3)	0.003 (3)
N8	0.039 (3)	0.036 (3)	0.045 (4)	−0.002 (2)	−0.007 (3)	0.000 (3)
N9	0.036 (3)	0.030 (3)	0.042 (4)	0.000 (2)	0.000 (3)	0.004 (2)
N10	0.052 (4)	0.034 (3)	0.038 (4)	−0.006 (3)	0.000 (3)	0.004 (2)
N11	0.047 (4)	0.028 (3)	0.046 (4)	−0.003 (2)	0.007 (3)	−0.002 (3)
N12	0.051 (4)	0.042 (3)	0.049 (4)	−0.003 (3)	0.011 (3)	−0.005 (3)
N13	0.039 (3)	0.036 (3)	0.037 (4)	−0.006 (2)	0.005 (3)	−0.003 (3)
N14	0.045 (3)	0.034 (3)	0.041 (4)	−0.004 (2)	0.003 (3)	−0.003 (3)
N15	0.039 (3)	0.034 (3)	0.050 (4)	−0.004 (3)	−0.004 (3)	0.006 (3)
N16	0.048 (4)	0.044 (3)	0.060 (4)	0.001 (3)	−0.013 (3)	0.001 (3)
N17	0.044 (4)	0.034 (3)	0.044 (4)	−0.002 (3)	−0.006 (3)	0.004 (3)
N18	0.045 (3)	0.038 (3)	0.044 (4)	−0.003 (2)	0.001 (3)	0.006 (3)
N19	0.040 (3)	0.030 (3)	0.050 (4)	−0.001 (2)	0.000 (3)	0.004 (3)
N20	0.046 (3)	0.039 (3)	0.044 (4)	0.003 (3)	0.002 (3)	0.001 (3)
N21	0.044 (3)	0.029 (3)	0.049 (4)	−0.004 (2)	0.004 (3)	−0.001 (3)
N22	0.037 (3)	0.032 (3)	0.056 (4)	−0.001 (2)	−0.001 (3)	−0.004 (3)
N23	0.031 (3)	0.043 (3)	0.050 (4)	0.001 (2)	−0.002 (3)	0.004 (3)
N24	0.039 (3)	0.048 (3)	0.076 (5)	−0.001 (3)	0.002 (3)	0.004 (3)
N25	0.050 (4)	0.036 (3)	0.033 (3)	0.003 (3)	−0.009 (3)	−0.004 (3)
N26	0.044 (3)	0.037 (3)	0.049 (4)	−0.001 (3)	−0.008 (3)	0.004 (3)
N27	0.038 (3)	0.040 (3)	0.039 (4)	0.007 (3)	0.001 (3)	0.000 (3)
N28	0.045 (3)	0.039 (3)	0.039 (4)	0.003 (3)	−0.005 (3)	0.002 (3)
N29	0.043 (3)	0.037 (3)	0.043 (4)	−0.002 (3)	0.009 (3)	0.002 (3)
N30	0.050 (4)	0.042 (3)	0.050 (4)	−0.002 (3)	0.010 (3)	0.001 (3)
N31	0.031 (3)	0.037 (3)	0.041 (4)	0.000 (2)	0.002 (3)	−0.005 (3)
N32	0.039 (3)	0.036 (3)	0.054 (4)	0.000 (3)	−0.002 (3)	−0.003 (3)
C1	0.040 (4)	0.064 (5)	0.061 (5)	0.005 (4)	−0.012 (4)	−0.017 (4)
C2	0.048 (5)	0.103 (7)	0.076 (7)	−0.012 (4)	−0.011 (5)	−0.011 (5)
C3	0.063 (5)	0.063 (5)	0.067 (6)	0.008 (4)	−0.021 (5)	0.002 (5)
C4	0.055 (5)	0.037 (4)	0.050 (5)	−0.004 (3)	−0.005 (4)	0.001 (3)
C5	0.081 (6)	0.062 (5)	0.057 (6)	−0.009 (4)	−0.019 (5)	0.009 (4)
C6	0.041 (4)	0.051 (4)	0.056 (5)	−0.007 (3)	0.002 (4)	−0.005 (4)
C7	0.064 (6)	0.063 (5)	0.093 (8)	0.004 (4)	0.014 (5)	−0.016 (5)
C8	0.070 (5)	0.056 (4)	0.047 (5)	−0.008 (4)	0.012 (4)	0.008 (4)
C9	0.049 (4)	0.043 (4)	0.038 (4)	−0.014 (3)	−0.009 (3)	0.001 (3)
C10	0.087 (6)	0.043 (4)	0.061 (6)	−0.007 (4)	−0.005 (5)	0.016 (4)
C11	0.083 (6)	0.055 (5)	0.055 (6)	−0.011 (4)	0.017 (5)	−0.010 (4)
C12	0.114 (8)	0.092 (7)	0.068 (7)	−0.018 (6)	0.024 (6)	−0.033 (5)
C13	0.072 (6)	0.062 (5)	0.061 (6)	−0.011 (4)	0.024 (5)	−0.005 (4)
C14	0.051 (5)	0.039 (4)	0.060 (5)	−0.003 (3)	0.008 (4)	−0.002 (4)
C15	0.050 (5)	0.057 (4)	0.089 (7)	−0.022 (4)	0.009 (5)	−0.013 (4)
C16	0.040 (4)	0.054 (4)	0.048 (5)	−0.007 (3)	−0.006 (4)	−0.003 (3)
C17	0.059 (6)	0.082 (6)	0.066 (7)	−0.014 (4)	−0.012 (5)	−0.018 (5)
C18	0.039 (4)	0.066 (5)	0.069 (6)	0.011 (4)	−0.009 (4)	0.008 (4)
C19	0.042 (4)	0.037 (4)	0.069 (6)	0.004 (3)	0.006 (4)	0.007 (4)
C20	0.055 (5)	0.041 (4)	0.126 (9)	0.011 (4)	0.002 (5)	−0.004 (5)
C21	0.057 (5)	0.047 (4)	0.042 (4)	0.003 (4)	−0.012 (4)	−0.004 (3)
C22	0.088 (6)	0.062 (5)	0.057 (6)	0.004 (4)	−0.025 (5)	0.014 (4)
C23	0.042 (4)	0.061 (5)	0.051 (5)	0.000 (4)	−0.009 (4)	−0.002 (4)
C24	0.041 (4)	0.039 (3)	0.048 (4)	0.000 (3)	0.002 (3)	−0.005 (3)
C25	0.046 (4)	0.059 (4)	0.076 (6)	−0.018 (3)	−0.002 (4)	0.016 (4)
C26	0.054 (5)	0.063 (5)	0.052 (5)	−0.005 (4)	0.015 (4)	−0.016 (4)
C27	0.109 (9)	0.095 (7)	0.107 (10)	0.024 (7)	0.043 (8)	−0.019 (6)
C28	0.078 (6)	0.071 (5)	0.044 (5)	−0.014 (5)	0.027 (5)	−0.009 (4)
C29	0.066 (5)	0.045 (4)	0.041 (5)	−0.018 (4)	0.000 (4)	−0.004 (3)
C30	0.114 (8)	0.050 (5)	0.049 (5)	−0.005 (5)	0.004 (5)	0.010 (4)
C31	0.059 (5)	0.045 (4)	0.043 (5)	0.006 (3)	0.013 (4)	0.004 (3)
C32	0.094 (7)	0.073 (5)	0.044 (5)	−0.006 (5)	0.013 (5)	−0.007 (4)
C33	0.057 (5)	0.060 (5)	0.059 (6)	−0.003 (4)	0.028 (4)	0.008 (4)
C34	0.041 (4)	0.038 (4)	0.059 (6)	−0.008 (3)	0.005 (4)	−0.003 (3)
C35	0.057 (5)	0.062 (5)	0.083 (7)	−0.020 (4)	0.020 (5)	−0.013 (4)
C36	0.056 (5)	0.052 (4)	0.077 (6)	0.000 (4)	−0.016 (5)	0.017 (4)
C37	0.088 (7)	0.077 (6)	0.131 (10)	0.029 (5)	−0.039 (7)	0.015 (6)
C38	0.078 (6)	0.065 (5)	0.052 (5)	−0.013 (4)	−0.027 (5)	0.007 (4)
C39	0.061 (5)	0.043 (4)	0.042 (5)	−0.009 (4)	−0.007 (4)	0.007 (3)
C40	0.111 (8)	0.050 (5)	0.060 (6)	−0.010 (5)	−0.014 (6)	−0.010 (4)
C41	0.049 (5)	0.060 (5)	0.040 (5)	0.008 (4)	−0.005 (4)	0.004 (4)
C42	0.070 (6)	0.090 (6)	0.073 (7)	0.012 (5)	−0.006 (5)	0.030 (5)
C43	0.040 (4)	0.072 (5)	0.055 (5)	−0.001 (4)	−0.007 (4)	0.001 (4)
C44	0.038 (4)	0.047 (4)	0.056 (5)	−0.005 (3)	0.001 (4)	−0.001 (3)
C45	0.058 (5)	0.051 (4)	0.100 (7)	−0.012 (4)	−0.010 (5)	0.004 (4)
C46	0.055 (5)	0.054 (4)	0.049 (5)	−0.009 (4)	−0.001 (4)	0.007 (4)
C47	0.098 (7)	0.086 (6)	0.050 (5)	−0.002 (5)	0.001 (5)	0.026 (5)
C48	0.044 (4)	0.063 (5)	0.062 (6)	−0.004 (4)	0.017 (4)	−0.002 (4)
C49	0.038 (4)	0.032 (3)	0.060 (5)	−0.004 (3)	0.004 (4)	−0.002 (3)
C50	0.043 (4)	0.050 (4)	0.104 (7)	0.006 (4)	0.017 (5)	0.004 (4)
C51	0.040 (4)	0.043 (4)	0.065 (6)	0.003 (3)	0.010 (4)	0.009 (4)
C52	0.059 (6)	0.073 (6)	0.084 (8)	−0.013 (4)	0.016 (5)	0.019 (5)
C53	0.046 (4)	0.061 (5)	0.048 (5)	0.005 (4)	0.005 (4)	0.000 (4)
C54	0.049 (4)	0.037 (4)	0.050 (5)	0.003 (3)	0.001 (4)	−0.004 (3)
C55	0.102 (7)	0.047 (5)	0.068 (7)	−0.017 (4)	0.009 (5)	−0.014 (4)
C56	0.047 (5)	0.061 (5)	0.099 (8)	−0.009 (4)	−0.026 (5)	0.032 (5)
C57	0.065 (6)	0.100 (7)	0.149 (11)	0.012 (6)	−0.035 (7)	0.023 (7)
C58	0.085 (7)	0.069 (6)	0.081 (7)	−0.019 (5)	−0.038 (6)	0.001 (5)
C59	0.058 (5)	0.046 (4)	0.055 (5)	−0.006 (4)	−0.017 (4)	0.003 (4)
C60	0.082 (6)	0.074 (6)	0.072 (7)	−0.002 (5)	−0.010 (5)	−0.016 (5)
C61	0.057 (5)	0.058 (5)	0.060 (5)	−0.001 (4)	−0.016 (4)	−0.013 (4)
C62	0.081 (7)	0.097 (7)	0.109 (9)	−0.038 (6)	−0.028 (6)	−0.012 (6)
C63	0.066 (5)	0.055 (5)	0.065 (6)	0.013 (4)	−0.037 (5)	−0.008 (4)
C64	0.066 (5)	0.042 (4)	0.042 (5)	0.012 (4)	−0.011 (4)	0.001 (3)
C65	0.122 (8)	0.053 (5)	0.055 (6)	0.002 (5)	−0.022 (6)	0.016 (4)
C66	0.055 (5)	0.041 (4)	0.046 (5)	−0.007 (3)	0.004 (4)	−0.011 (3)
C67	0.080 (6)	0.076 (5)	0.047 (5)	−0.006 (4)	0.003 (4)	0.004 (4)
C68	0.044 (4)	0.064 (5)	0.056 (5)	0.000 (4)	0.011 (4)	−0.014 (4)
C69	0.041 (4)	0.036 (4)	0.050 (5)	0.001 (3)	0.001 (4)	−0.005 (3)
C70	0.057 (5)	0.057 (5)	0.086 (7)	0.021 (4)	0.001 (5)	0.005 (4)
C71	0.063 (5)	0.046 (4)	0.040 (5)	0.010 (4)	0.004 (4)	0.002 (3)
C72	0.119 (8)	0.054 (5)	0.066 (6)	−0.005 (5)	0.012 (6)	−0.009 (5)
C73	0.078 (6)	0.069 (5)	0.043 (5)	0.010 (4)	0.017 (5)	0.003 (4)
C74	0.057 (5)	0.056 (5)	0.061 (6)	0.007 (4)	0.016 (4)	0.011 (4)
C75	0.078 (7)	0.084 (6)	0.096 (8)	−0.021 (5)	0.030 (6)	0.017 (5)
C76	0.050 (5)	0.048 (4)	0.059 (5)	−0.012 (4)	−0.007 (4)	−0.004 (4)
C77	0.073 (7)	0.072 (6)	0.126 (9)	−0.020 (5)	−0.015 (6)	−0.033 (6)
C78	0.040 (4)	0.059 (5)	0.065 (6)	0.002 (4)	−0.012 (4)	0.003 (4)
C79	0.038 (4)	0.041 (4)	0.055 (5)	0.005 (3)	0.002 (4)	0.005 (3)
C80	0.051 (5)	0.058 (4)	0.095 (7)	0.018 (3)	0.003 (5)	−0.012 (5)

### Geometric parameters (Å, º)

**Table:**

Cu1—O1	2.289 (4)	C17—H17B	0.9600
Cu1—O14^i^	2.438 (4)	C17—H17C	0.9600
Cu1—N1	2.039 (6)	C18—H18A	0.9300
Cu1—N3	2.054 (6)	C18—C19	1.379 (11)
Cu1—N5	2.033 (6)	C19—C20	1.510 (10)
Cu1—N7	2.028 (6)	C20—H20A	0.9600
Cu2—O2	2.395 (4)	C20—H20B	0.9602
Cu2—O5	2.341 (4)	C20—H20C	0.9599
Cu2—N9	2.033 (6)	C21—C22	1.504 (10)
Cu2—N11	2.042 (6)	C21—C23	1.358 (10)
Cu2—N13	2.031 (6)	C22—H22A	0.9600
Cu2—N15	2.028 (6)	C22—H22B	0.9600
Cu3—O6	2.367 (4)	C22—H22C	0.9600
Cu3—O9	2.341 (4)	C23—H23	0.9300
Cu3—N17	2.041 (6)	C23—C24	1.401 (10)
Cu3—N19	2.044 (6)	C24—C25	1.487 (9)
Cu3—N21	2.040 (6)	C25—H25A	0.9600
Cu3—N23	2.058 (6)	C25—H25B	0.9600
Cu4—O10	2.435 (4)	C25—H25C	0.9600
Cu4—O13	2.339 (4)	C26—C27	1.504 (11)
Cu4—N25	2.014 (6)	C26—C28	1.364 (11)
Cu4—N27	2.045 (6)	C27—H27A	0.9602
Cu4—N29	2.031 (6)	C27—H27B	0.9598
Cu4—N31	2.030 (6)	C27—H27C	0.9597
S1—O1	1.468 (5)	C28—H28A	0.9300
S1—O2	1.465 (4)	C28—C29	1.403 (11)
S1—O3	1.477 (6)	C29—C30	1.493 (10)
S1—O4	1.462 (5)	C30—H30A	0.9600
S2—O5	1.465 (5)	C30—H30B	0.9600
S2—O6	1.456 (5)	C30—H30C	0.9600
S2—O7	1.475 (5)	C31—C32	1.504 (10)
S2—O8	1.463 (6)	C31—C33	1.369 (10)
S3—O9	1.468 (5)	C32—H32A	0.9599
S3—O10	1.463 (5)	C32—H32B	0.9602
S3—O11	1.465 (6)	C32—H32C	0.9600
S3—O12	1.465 (5)	C33—H33	0.9300
S4—O13	1.475 (5)	C33—C34	1.398 (11)
S4—O14	1.466 (5)	C34—C35	1.492 (10)
S4—O15	1.459 (6)	C35—H35A	0.9600
S4—O16	1.473 (5)	C35—H35B	0.9600
N1—N2	1.373 (7)	C35—H35C	0.9599
N1—C4	1.339 (9)	C36—C37	1.506 (10)
N2—H2	0.8600	C36—C38	1.374 (11)
N2—C1	1.338 (9)	C37—H37A	0.9596
N3—N4	1.361 (7)	C37—H37B	0.9601
N3—C9	1.338 (9)	C37—H37C	0.9604
N4—H4	0.8600	C38—H38	0.9300
N4—C6	1.325 (9)	C38—C39	1.387 (11)
N5—N6	1.351 (8)	C39—C40	1.477 (11)
N5—C14	1.325 (9)	C40—H40A	0.9601
N6—H6	0.8600	C40—H40B	0.9599
N6—C11	1.343 (10)	C40—H40C	0.9598
N7—N8	1.365 (7)	C41—C42	1.500 (10)
N7—C19	1.325 (9)	C41—C43	1.364 (10)
N8—H8	0.8600	C42—H42A	0.9600
N8—C16	1.341 (9)	C42—H42B	0.9600
N9—N10	1.358 (7)	C42—H42C	0.9600
N9—C24	1.337 (8)	C43—H43	0.9300
N10—H10	0.8600	C43—C44	1.395 (10)
N10—C21	1.326 (9)	C44—C45	1.505 (10)
N11—N12	1.347 (7)	C45—H45A	0.9601
N11—C29	1.335 (9)	C45—H45B	0.9600
N12—H12	0.8600	C45—H45C	0.9601
N12—C26	1.352 (9)	C46—C47	1.503 (10)
N13—N14	1.347 (7)	C46—C48	1.371 (10)
N13—C34	1.345 (8)	C47—H47A	0.9598
N14—H14	0.8600	C47—H47B	0.9607
N14—C31	1.322 (9)	C47—H47C	0.9599
N15—N16	1.362 (7)	C48—H48	0.9300
N15—C39	1.333 (9)	C48—C49	1.387 (11)
N16—H16	0.8600	C49—C50	1.495 (10)
N16—C36	1.326 (9)	C50—H50A	0.9600
N17—N18	1.341 (7)	C50—H50B	0.9600
N17—C44	1.341 (8)	C50—H50C	0.9600
N18—H18	0.8600	C51—C52	1.499 (10)
N18—C41	1.327 (9)	C51—C53	1.372 (10)
N19—N20	1.355 (7)	C52—H52A	0.9600
N19—C49	1.315 (9)	C52—H52B	0.9599
N20—H20	0.8600	C52—H52C	0.9609
N20—C46	1.318 (9)	C53—H53	0.9300
N21—N22	1.355 (7)	C53—C54	1.371 (10)
N21—C54	1.342 (9)	C54—C55	1.524 (10)
N22—H22	0.8600	C55—H55A	0.9600
N22—C51	1.316 (9)	C55—H55B	0.9600
N23—N24	1.346 (7)	C55—H55C	0.9600
N23—C59	1.324 (10)	C56—C57	1.512 (11)
N24—H24	0.8600	C56—C58	1.387 (13)
N24—C56	1.341 (10)	C57—H57A	0.9605
N25—N26	1.344 (7)	C57—H57B	0.9602
N25—C64	1.355 (9)	C57—H57C	0.9599
N26—H26	0.8600	C58—H58	0.9300
N26—C61	1.340 (9)	C58—C59	1.371 (11)
N27—N28	1.373 (7)	C59—C60	1.494 (11)
N27—C69	1.331 (8)	C60—H60A	0.9599
N28—H28	0.8600	C60—H60B	0.9604
N28—C66	1.327 (9)	C60—H60C	0.9597
N29—N30	1.363 (7)	C61—C62	1.512 (11)
N29—C71	1.319 (9)	C61—C63	1.355 (11)
N30—H30	0.8600	C62—H62A	0.9600
N30—C74	1.332 (10)	C62—H62B	0.9600
N31—N32	1.350 (7)	C62—H62C	0.9600
N31—C79	1.354 (8)	C63—H63	0.9300
N32—H32	0.8600	C63—C64	1.396 (10)
N32—C76	1.341 (9)	C64—C65	1.490 (11)
C1—C2	1.490 (10)	C65—H65A	0.9599
C1—C3	1.364 (11)	C65—H65B	0.9603
C2—H2A	0.9605	C65—H65C	0.9604
C2—H2B	0.9601	C66—C67	1.453 (10)
C2—H2C	0.9600	C66—C68	1.382 (10)
C3—H3	0.9300	C67—H67A	0.9600
C3—C4	1.400 (11)	C67—H67B	0.9599
C4—C5	1.482 (10)	C67—H67C	0.9605
C5—H5A	0.9599	C68—H68	0.9300
C5—H5B	0.9605	C68—C69	1.369 (11)
C5—H5C	0.9609	C69—C70	1.492 (10)
C6—C7	1.504 (10)	C70—H70A	0.9603
C6—C8	1.366 (10)	C70—H70B	0.9599
C7—H7A	0.9604	C70—H70C	0.9599
C7—H7B	0.9602	C71—C72	1.516 (11)
C7—H7C	0.9599	C71—C73	1.385 (10)
C8—H8A	0.9300	C72—H72A	0.9603
C8—C9	1.386 (10)	C72—H72B	0.9601
C9—C10	1.482 (10)	C72—H72C	0.9600
C10—H10A	0.9600	C73—H73	0.9300
C10—H10B	0.9600	C73—C74	1.369 (11)
C10—H10C	0.9600	C74—C75	1.501 (11)
C11—C12	1.505 (11)	C75—H75A	0.9600
C11—C13	1.368 (11)	C75—H75B	0.9596
C12—H12A	0.9596	C75—H75C	0.9600
C12—H12B	0.9600	C76—C77	1.505 (11)
C12—H12C	0.9604	C76—C78	1.365 (10)
C13—H13	0.9300	C77—H77A	0.9602
C13—C14	1.381 (11)	C77—H77B	0.9601
C14—C15	1.502 (10)	C77—H77C	0.9598
C15—H15A	0.9599	C78—H78	0.9300
C15—H15B	0.9606	C78—C79	1.393 (10)
C15—H15C	0.9597	C79—C80	1.490 (9)
C16—C17	1.495 (11)	C80—H80A	0.9599
C16—C18	1.378 (10)	C80—H80B	0.9599
C17—H17A	0.9600	C80—H80C	0.9600
			
O1—Cu1—O14^i^	179.5 (2)	C16—C18—C19	106.3 (6)
N1—Cu1—O1	90.64 (19)	C19—C18—H18A	126.8
N1—Cu1—O14^i^	88.90 (19)	N7—C19—C18	111.0 (6)
N1—Cu1—N3	88.0 (2)	N7—C19—C20	120.5 (7)
N3—Cu1—O1	91.7 (2)	C18—C19—C20	128.5 (7)
N3—Cu1—O14^i^	88.2 (2)	C19—C20—H20A	109.4
N5—Cu1—O1	86.5 (2)	C19—C20—H20B	109.5
N5—Cu1—O14^i^	93.6 (2)	C19—C20—H20C	109.5
N5—Cu1—N1	90.6 (2)	H20A—C20—H20B	109.5
N5—Cu1—N3	177.7 (2)	H20A—C20—H20C	109.5
N7—Cu1—O1	96.5 (2)	H20B—C20—H20C	109.5
N7—Cu1—O14^i^	83.98 (19)	N10—C21—C22	120.4 (7)
N7—Cu1—N1	172.8 (2)	N10—C21—C23	106.4 (6)
N7—Cu1—N3	90.4 (2)	C23—C21—C22	133.2 (7)
N7—Cu1—N5	91.2 (2)	C21—C22—H22A	109.5
O5—Cu2—O2	178.8 (2)	C21—C22—H22B	109.5
N9—Cu2—O2	89.05 (19)	C21—C22—H22C	109.5
N9—Cu2—O5	89.76 (19)	H22A—C22—H22B	109.5
N9—Cu2—N11	87.6 (2)	H22A—C22—H22C	109.5
N11—Cu2—O2	84.0 (2)	H22B—C22—H22C	109.5
N11—Cu2—O5	96.0 (2)	C21—C23—H23	126.8
N13—Cu2—O2	89.1 (2)	C21—C23—C24	106.4 (6)
N13—Cu2—O5	90.9 (2)	C24—C23—H23	126.8
N13—Cu2—N9	89.1 (2)	N9—C24—C23	109.7 (6)
N13—Cu2—N11	172.4 (2)	N9—C24—C25	123.6 (6)
N15—Cu2—O2	94.08 (19)	C23—C24—C25	126.6 (7)
N15—Cu2—O5	87.11 (19)	C24—C25—H25A	109.5
N15—Cu2—N9	176.5 (2)	C24—C25—H25B	109.5
N15—Cu2—N11	91.2 (2)	C24—C25—H25C	109.5
N15—Cu2—N13	92.4 (2)	H25A—C25—H25B	109.5
O9—Cu3—O6	177.2 (3)	H25A—C25—H25C	109.5
N17—Cu3—O6	85.2 (2)	H25B—C25—H25C	109.5
N17—Cu3—O9	97.2 (2)	N12—C26—C27	119.2 (8)
N17—Cu3—N19	89.8 (2)	N12—C26—C28	107.0 (7)
N17—Cu3—N23	173.2 (2)	C28—C26—C27	133.8 (8)
N19—Cu3—O6	92.5 (2)	C26—C27—H27A	109.7
N19—Cu3—O9	86.0 (2)	C26—C27—H27B	109.6
N19—Cu3—N23	90.1 (2)	C26—C27—H27C	109.1
N21—Cu3—O6	91.7 (2)	H27A—C27—H27B	109.5
N21—Cu3—O9	89.9 (2)	H27A—C27—H27C	109.5
N21—Cu3—N17	89.4 (2)	H27B—C27—H27C	109.5
N21—Cu3—N19	175.7 (2)	C26—C28—H28A	126.8
N21—Cu3—N23	91.3 (2)	C26—C28—C29	106.3 (7)
N23—Cu3—O6	88.1 (2)	C29—C28—H28A	126.8
N23—Cu3—O9	89.6 (2)	N11—C29—C28	109.0 (7)
O13—Cu4—O10	178.39 (17)	N11—C29—C30	122.0 (7)
N25—Cu4—O10	96.2 (2)	C28—C29—C30	129.0 (7)
N25—Cu4—O13	84.87 (19)	C29—C30—H30A	109.5
N25—Cu4—N27	89.7 (2)	C29—C30—H30B	109.5
N25—Cu4—N29	91.8 (2)	C29—C30—H30C	109.5
N25—Cu4—N31	175.6 (2)	H30A—C30—H30B	109.5
N27—Cu4—O10	88.1 (2)	H30A—C30—H30C	109.5
N27—Cu4—O13	93.1 (2)	H30B—C30—H30C	109.5
N29—Cu4—O10	83.4 (2)	N14—C31—C32	121.3 (7)
N29—Cu4—O13	95.3 (2)	N14—C31—C33	105.4 (7)
N29—Cu4—N27	171.5 (2)	C33—C31—C32	133.2 (7)
N31—Cu4—O10	88.2 (2)	C31—C32—H32A	109.3
N31—Cu4—O13	90.79 (19)	C31—C32—H32B	109.6
N31—Cu4—N27	89.9 (2)	C31—C32—H32C	109.5
N31—Cu4—N29	89.2 (2)	H32A—C32—H32B	109.5
O1—S1—O3	108.0 (3)	H32A—C32—H32C	109.5
O2—S1—O1	110.5 (3)	H32B—C32—H32C	109.5
O2—S1—O3	109.7 (4)	C31—C33—H33	126.5
O4—S1—O1	110.5 (3)	C31—C33—C34	107.0 (7)
O4—S1—O2	108.0 (3)	C34—C33—H33	126.5
O4—S1—O3	110.1 (4)	N13—C34—C33	109.0 (6)
O5—S2—O7	108.4 (3)	N13—C34—C35	123.4 (7)
O6—S2—O5	110.2 (3)	C33—C34—C35	127.6 (7)
O6—S2—O7	109.1 (3)	C34—C35—H35A	109.4
O6—S2—O8	108.4 (3)	C34—C35—H35B	109.4
O8—S2—O5	110.2 (3)	C34—C35—H35C	109.6
O8—S2—O7	110.4 (4)	H35A—C35—H35B	109.5
O10—S3—O9	109.5 (3)	H35A—C35—H35C	109.5
O10—S3—O11	109.3 (4)	H35B—C35—H35C	109.5
O10—S3—O12	108.3 (3)	N16—C36—C37	122.1 (8)
O11—S3—O9	108.7 (4)	N16—C36—C38	106.4 (7)
O11—S3—O12	110.5 (4)	C38—C36—C37	131.5 (9)
O12—S3—O9	110.4 (3)	C36—C37—H37A	109.4
O14—S4—O13	110.2 (3)	C36—C37—H37B	109.6
O14—S4—O16	109.1 (3)	C36—C37—H37C	109.5
O15—S4—O13	110.9 (3)	H37A—C37—H37B	109.5
O15—S4—O14	108.6 (3)	H37A—C37—H37C	109.5
O15—S4—O16	110.3 (4)	H37B—C37—H37C	109.4
O16—S4—O13	107.7 (3)	C36—C38—H38	126.4
S1—O1—Cu1	141.5 (3)	C36—C38—C39	107.2 (7)
S1—O2—Cu2	150.2 (3)	C39—C38—H38	126.4
S2—O5—Cu2	140.8 (3)	N15—C39—C38	108.7 (7)
S2—O6—Cu3	146.0 (3)	N15—C39—C40	122.1 (7)
S3—O9—Cu3	143.0 (3)	C38—C39—C40	129.2 (8)
S3—O10—Cu4	148.4 (4)	C39—C40—H40A	109.4
S4—O13—Cu4	138.1 (3)	C39—C40—H40B	109.5
Cu1^ii^—O14—Cu1^i^	175.78 (12)	C39—C40—H40C	109.5
S4—O14—Cu1^i^	26.2 (2)	H40A—C40—H40B	109.5
S4—O14—Cu1^ii^	151.4 (3)	H40A—C40—H40C	109.5
N2—N1—Cu1	119.1 (4)	H40B—C40—H40C	109.5
C4—N1—Cu1	135.0 (5)	N18—C41—C42	122.1 (7)
C4—N1—N2	105.4 (6)	N18—C41—C43	105.9 (6)
N1—N2—H2	124.3	C43—C41—C42	132.0 (8)
C1—N2—N1	111.5 (6)	C41—C42—H42A	109.5
C1—N2—H2	124.3	C41—C42—H42B	109.5
N4—N3—Cu1	117.0 (4)	C41—C42—H42C	109.5
C9—N3—Cu1	137.1 (5)	H42A—C42—H42B	109.5
C9—N3—N4	105.9 (6)	H42A—C42—H42C	109.5
N3—N4—H4	124.2	H42B—C42—H42C	109.5
C6—N4—N3	111.6 (6)	C41—C43—H43	126.7
C6—N4—H4	124.2	C41—C43—C44	106.7 (7)
N6—N5—Cu1	116.9 (4)	C44—C43—H43	126.7
C14—N5—Cu1	137.0 (5)	N17—C44—C43	109.1 (6)
C14—N5—N6	105.7 (6)	N17—C44—C45	122.3 (7)
N5—N6—H6	123.9	C43—C44—C45	128.6 (7)
C11—N6—N5	112.3 (6)	C44—C45—H45A	109.4
C11—N6—H6	123.9	C44—C45—H45B	109.5
N8—N7—Cu1	117.5 (4)	C44—C45—H45C	109.5
C19—N7—Cu1	137.5 (5)	H45A—C45—H45B	109.5
C19—N7—N8	104.8 (6)	H45A—C45—H45C	109.5
N7—N8—H8	124.0	H45B—C45—H45C	109.5
C16—N8—N7	112.0 (6)	N20—C46—C47	122.3 (7)
C16—N8—H8	124.0	N20—C46—C48	106.4 (7)
N10—N9—Cu2	118.5 (4)	C48—C46—C47	131.2 (8)
C24—N9—Cu2	135.4 (5)	C46—C47—H47A	109.5
C24—N9—N10	104.5 (5)	C46—C47—H47B	109.4
N9—N10—H10	123.6	C46—C47—H47C	109.6
C21—N10—N9	112.9 (6)	H47A—C47—H47B	109.4
C21—N10—H10	123.6	H47A—C47—H47C	109.5
N12—N11—Cu2	116.3 (4)	H47B—C47—H47C	109.4
C29—N11—Cu2	136.8 (5)	C46—C48—H48	126.9
C29—N11—N12	106.9 (6)	C46—C48—C49	106.2 (7)
N11—N12—H12	124.6	C49—C48—H48	126.9
N11—N12—C26	110.8 (6)	N19—C49—C48	109.7 (6)
C26—N12—H12	124.6	N19—C49—C50	121.5 (7)
N14—N13—Cu2	118.9 (4)	C48—C49—C50	128.8 (7)
C34—N13—Cu2	135.7 (5)	C49—C50—H50A	109.5
C34—N13—N14	104.7 (6)	C49—C50—H50B	109.5
N13—N14—H14	123.1	C49—C50—H50C	109.5
C31—N14—N13	113.9 (6)	H50A—C50—H50B	109.5
C31—N14—H14	123.1	H50A—C50—H50C	109.5
N16—N15—Cu2	117.2 (5)	H50B—C50—H50C	109.5
C39—N15—Cu2	136.0 (5)	N22—C51—C52	122.4 (7)
C39—N15—N16	106.5 (6)	N22—C51—C53	106.0 (6)
N15—N16—H16	124.4	C53—C51—C52	131.5 (8)
C36—N16—N15	111.3 (6)	C51—C52—H52A	109.4
C36—N16—H16	124.4	C51—C52—H52B	109.5
N18—N17—Cu3	119.4 (4)	C51—C52—H52C	109.5
C44—N17—Cu3	135.2 (5)	H52A—C52—H52B	109.5
C44—N17—N18	105.1 (6)	H52A—C52—H52C	109.5
N17—N18—H18	123.4	H52B—C52—H52C	109.5
C41—N18—N17	113.1 (6)	C51—C53—H53	126.9
C41—N18—H18	123.4	C51—C53—C54	106.3 (7)
N20—N19—Cu3	116.8 (4)	C54—C53—H53	126.9
C49—N19—Cu3	137.3 (5)	N21—C54—C53	110.6 (6)
C49—N19—N20	105.9 (6)	N21—C54—C55	121.8 (7)
N19—N20—H20	124.1	C53—C54—C55	127.6 (7)
C46—N20—N19	111.8 (6)	C54—C55—H55A	109.5
C46—N20—H20	124.1	C54—C55—H55B	109.5
N22—N21—Cu3	119.5 (4)	C54—C55—H55C	109.5
C54—N21—Cu3	135.7 (5)	H55A—C55—H55B	109.5
C54—N21—N22	103.7 (6)	H55A—C55—H55C	109.5
N21—N22—H22	123.4	H55B—C55—H55C	109.5
C51—N22—N21	113.2 (6)	N24—C56—C57	121.8 (10)
C51—N22—H22	123.4	N24—C56—C58	105.5 (7)
N24—N23—Cu3	119.3 (5)	C58—C56—C57	132.6 (9)
C59—N23—Cu3	135.0 (5)	C56—C57—H57A	109.4
C59—N23—N24	105.7 (6)	C56—C57—H57B	109.4
N23—N24—H24	124.0	C56—C57—H57C	109.7
C56—N24—N23	111.9 (7)	H57A—C57—H57B	109.5
C56—N24—H24	124.0	H57A—C57—H57C	109.4
N26—N25—Cu4	119.4 (4)	H57B—C57—H57C	109.5
N26—N25—C64	105.1 (6)	C56—C58—H58	126.9
C64—N25—Cu4	135.5 (5)	C59—C58—C56	106.2 (8)
N25—N26—H26	123.6	C59—C58—H58	126.9
C61—N26—N25	112.8 (6)	N23—C59—C58	110.7 (8)
C61—N26—H26	123.6	N23—C59—C60	123.7 (7)
N28—N27—Cu4	118.3 (4)	C58—C59—C60	125.6 (8)
C69—N27—Cu4	135.8 (5)	C59—C60—H60A	109.4
C69—N27—N28	105.9 (6)	C59—C60—H60B	109.5
N27—N28—H28	123.9	C59—C60—H60C	109.5
C66—N28—N27	112.2 (6)	H60A—C60—H60B	109.5
C66—N28—H28	123.9	H60A—C60—H60C	109.5
N30—N29—Cu4	117.1 (4)	H60B—C60—H60C	109.5
C71—N29—Cu4	138.3 (5)	N26—C61—C62	121.3 (8)
C71—N29—N30	104.5 (6)	N26—C61—C63	105.9 (7)
N29—N30—H30	124.0	C63—C61—C62	132.8 (8)
C74—N30—N29	112.0 (6)	C61—C62—H62A	109.5
C74—N30—H30	124.0	C61—C62—H62B	109.5
N32—N31—Cu4	119.4 (4)	C61—C62—H62C	109.5
N32—N31—C79	105.4 (5)	H62A—C62—H62B	109.5
C79—N31—Cu4	134.2 (5)	H62A—C62—H62C	109.5
N31—N32—H32	123.9	H62B—C62—H62C	109.5
C76—N32—N31	112.3 (6)	C61—C63—H63	126.2
C76—N32—H32	123.9	C61—C63—C64	107.6 (7)
N2—C1—C2	119.3 (8)	C64—C63—H63	126.2
N2—C1—C3	106.9 (7)	N25—C64—C63	108.6 (7)
C3—C1—C2	133.8 (8)	N25—C64—C65	121.0 (7)
C1—C2—H2A	109.3	C63—C64—C65	130.4 (7)
C1—C2—H2B	109.4	C64—C65—H65A	109.6
C1—C2—H2C	109.6	C64—C65—H65B	109.5
H2A—C2—H2B	109.5	C64—C65—H65C	109.4
H2A—C2—H2C	109.5	H65A—C65—H65B	109.5
H2B—C2—H2C	109.5	H65A—C65—H65C	109.5
C1—C3—H3	126.6	H65B—C65—H65C	109.5
C1—C3—C4	106.8 (7)	N28—C66—C67	123.4 (7)
C4—C3—H3	126.6	N28—C66—C68	104.4 (7)
N1—C4—C3	109.5 (7)	C68—C66—C67	132.1 (8)
N1—C4—C5	123.2 (7)	C66—C67—H67A	109.7
C3—C4—C5	127.3 (8)	C66—C67—H67B	109.4
C4—C5—H5A	109.4	C66—C67—H67C	109.4
C4—C5—H5B	109.6	H67A—C67—H67B	109.5
C4—C5—H5C	109.6	H67A—C67—H67C	109.5
H5A—C5—H5B	109.5	H67B—C67—H67C	109.5
H5A—C5—H5C	109.5	C66—C68—H68	125.5
H5B—C5—H5C	109.3	C69—C68—C66	109.1 (7)
N4—C6—C7	121.2 (7)	C69—C68—H68	125.5
N4—C6—C8	106.6 (7)	N27—C69—C68	108.4 (7)
C8—C6—C7	132.1 (8)	N27—C69—C70	121.9 (7)
C6—C7—H7A	109.4	C68—C69—C70	129.7 (7)
C6—C7—H7B	109.6	C69—C70—H70A	109.4
C6—C7—H7C	109.5	C69—C70—H70B	109.4
H7A—C7—H7B	109.4	C69—C70—H70C	109.6
H7A—C7—H7C	109.5	H70A—C70—H70B	109.5
H7B—C7—H7C	109.5	H70A—C70—H70C	109.5
C6—C8—H8A	126.4	H70B—C70—H70C	109.5
C6—C8—C9	107.2 (7)	N29—C71—C72	120.3 (7)
C9—C8—H8A	126.4	N29—C71—C73	111.4 (7)
N3—C9—C8	108.8 (6)	C73—C71—C72	128.3 (7)
N3—C9—C10	122.6 (7)	C71—C72—H72A	109.5
C8—C9—C10	128.6 (7)	C71—C72—H72B	109.5
C9—C10—H10A	109.1	C71—C72—H72C	109.5
C9—C10—H10B	109.8	H72A—C72—H72B	109.4
C9—C10—H10C	109.6	H72A—C72—H72C	109.5
H10A—C10—H10B	109.5	H72B—C72—H72C	109.5
H10A—C10—H10C	109.5	C71—C73—H73	127.2
H10B—C10—H10C	109.5	C74—C73—C71	105.6 (7)
N6—C11—C12	121.8 (7)	C74—C73—H73	127.2
N6—C11—C13	104.9 (7)	N30—C74—C73	106.6 (7)
C13—C11—C12	133.3 (8)	N30—C74—C75	121.0 (8)
C11—C12—H12A	109.6	C73—C74—C75	132.4 (8)
C11—C12—H12B	109.4	C74—C75—H75A	109.4
C11—C12—H12C	109.5	C74—C75—H75B	109.7
H12A—C12—H12B	109.5	C74—C75—H75C	109.3
H12A—C12—H12C	109.5	H75A—C75—H75B	109.5
H12B—C12—H12C	109.4	H75A—C75—H75C	109.5
C11—C13—H13	126.2	H75B—C75—H75C	109.5
C11—C13—C14	107.7 (7)	N32—C76—C77	121.2 (7)
C14—C13—H13	126.2	N32—C76—C78	106.2 (6)
N5—C14—C13	109.4 (7)	C78—C76—C77	132.6 (8)
N5—C14—C15	122.5 (7)	C76—C77—H77A	109.4
C13—C14—C15	128.1 (7)	C76—C77—H77B	109.5
C14—C15—H15A	109.3	C76—C77—H77C	109.6
C14—C15—H15B	109.6	H77A—C77—H77B	109.4
C14—C15—H15C	109.5	H77A—C77—H77C	109.5
H15A—C15—H15B	109.5	H77B—C77—H77C	109.5
H15A—C15—H15C	109.5	C76—C78—H78	126.4
H15B—C15—H15C	109.4	C76—C78—C79	107.1 (7)
N8—C16—C17	121.2 (7)	C79—C78—H78	126.4
N8—C16—C18	105.8 (6)	N31—C79—C78	108.9 (6)
C18—C16—C17	133.0 (7)	N31—C79—C80	122.6 (6)
C16—C17—H17A	109.5	C78—C79—C80	128.5 (7)
C16—C17—H17B	109.5	C79—C80—H80A	109.5
C16—C17—H17C	109.5	C79—C80—H80B	109.5
H17A—C17—H17B	109.5	C79—C80—H80C	109.3
H17A—C17—H17C	109.5	H80A—C80—H80B	109.5
H17B—C17—H17C	109.5	H80A—C80—H80C	109.5
C16—C18—H18A	126.8	H80B—C80—H80C	109.5
			
Cu1—N1—N2—C1	−174.0 (5)	N15—N16—C36—C37	−178.2 (8)
Cu1—N1—C4—C3	172.4 (6)	N15—N16—C36—C38	1.6 (9)
Cu1—N1—C4—C5	−9.5 (12)	N16—N15—C39—C38	1.3 (8)
Cu1—N3—N4—C6	178.9 (5)	N16—N15—C39—C40	−179.1 (7)
Cu1—N3—C9—C8	−179.3 (5)	N16—C36—C38—C39	−0.7 (9)
Cu1—N3—C9—C10	0.8 (11)	N17—N18—C41—C42	−178.0 (7)
Cu1—N5—N6—C11	−175.4 (5)	N17—N18—C41—C43	0.4 (9)
Cu1—N5—C14—C13	173.2 (6)	N18—N17—C44—C43	−0.1 (8)
Cu1—N5—C14—C15	−7.5 (12)	N18—N17—C44—C45	−180.0 (7)
Cu1—N7—N8—C16	175.9 (5)	N18—C41—C43—C44	−0.4 (9)
Cu1—N7—C19—C18	−174.7 (6)	N19—N20—C46—C47	−176.9 (7)
Cu1—N7—C19—C20	5.7 (12)	N19—N20—C46—C48	1.2 (8)
Cu2—N9—N10—C21	166.0 (5)	N20—N19—C49—C48	1.9 (8)
Cu2—N9—C24—C23	−164.1 (5)	N20—N19—C49—C50	179.4 (7)
Cu2—N9—C24—C25	16.4 (11)	N20—C46—C48—C49	0.0 (9)
Cu2—N11—N12—C26	−176.7 (5)	N21—N22—C51—C52	−175.8 (7)
Cu2—N11—C29—C28	176.4 (6)	N21—N22—C51—C53	2.0 (8)
Cu2—N11—C29—C30	−2.8 (12)	N22—N21—C54—C53	−0.7 (8)
Cu2—N13—N14—C31	−173.2 (5)	N22—N21—C54—C55	178.3 (7)
Cu2—N13—C34—C33	170.5 (5)	N22—C51—C53—C54	−2.3 (8)
Cu2—N13—C34—C35	−11.9 (11)	N23—N24—C56—C57	−177.0 (7)
Cu2—N15—N16—C36	173.1 (5)	N23—N24—C56—C58	1.5 (9)
Cu2—N15—C39—C38	−172.1 (6)	N24—N23—C59—C58	0.4 (9)
Cu2—N15—C39—C40	7.4 (12)	N24—N23—C59—C60	179.1 (7)
Cu3—N17—N18—C41	174.2 (5)	N24—C56—C58—C59	−1.2 (10)
Cu3—N17—C44—C43	−173.2 (5)	N25—N26—C61—C62	178.9 (7)
Cu3—N17—C44—C45	6.9 (12)	N25—N26—C61—C63	0.8 (9)
Cu3—N19—N20—C46	−179.8 (5)	N26—N25—C64—C63	1.0 (8)
Cu3—N19—C49—C48	179.1 (5)	N26—N25—C64—C65	−179.7 (7)
Cu3—N19—C49—C50	−3.5 (12)	N26—C61—C63—C64	−0.1 (9)
Cu3—N21—N22—C51	169.4 (5)	N27—N28—C66—C67	−175.1 (7)
Cu3—N21—C54—C53	−168.5 (5)	N27—N28—C66—C68	1.9 (8)
Cu3—N21—C54—C55	10.6 (11)	N28—N27—C69—C68	0.3 (8)
Cu3—N23—N24—C56	176.5 (5)	N28—N27—C69—C70	179.0 (6)
Cu3—N23—C59—C58	−176.8 (6)	N28—C66—C68—C69	−1.6 (8)
Cu3—N23—C59—C60	1.9 (12)	N29—N30—C74—C73	1.0 (9)
Cu4—N25—N26—C61	−178.7 (5)	N29—N30—C74—C75	179.5 (7)
Cu4—N25—C64—C63	178.0 (5)	N29—C71—C73—C74	0.9 (9)
Cu4—N25—C64—C65	−2.7 (12)	N30—N29—C71—C72	179.5 (7)
Cu4—N27—N28—C66	178.2 (4)	N30—N29—C71—C73	−0.3 (8)
Cu4—N27—C69—C68	−179.2 (5)	N31—N32—C76—C77	178.7 (8)
Cu4—N27—C69—C70	−0.5 (11)	N31—N32—C76—C78	0.2 (9)
Cu4—N29—N30—C74	−177.8 (5)	N32—N31—C79—C78	0.8 (8)
Cu4—N29—C71—C72	−4.0 (12)	N32—N31—C79—C80	−178.5 (7)
Cu4—N29—C71—C73	176.2 (6)	N32—C76—C78—C79	0.4 (9)
Cu4—N31—N32—C76	169.6 (5)	C1—C3—C4—N1	−0.8 (9)
Cu4—N31—C79—C78	−167.3 (5)	C1—C3—C4—C5	−178.7 (8)
Cu4—N31—C79—C80	13.3 (11)	C2—C1—C3—C4	178.7 (9)
O1—S1—O2—Cu2	170.6 (6)	C4—N1—N2—C1	−1.0 (7)
O2—S1—O1—Cu1	177.8 (5)	C6—C8—C9—N3	0.5 (9)
O3—S1—O1—Cu1	−62.2 (7)	C6—C8—C9—C10	−179.6 (7)
O3—S1—O2—Cu2	51.6 (8)	C7—C6—C8—C9	176.2 (8)
O4—S1—O1—Cu1	58.3 (7)	C9—N3—N4—C6	−0.5 (7)
O4—S1—O2—Cu2	−68.4 (8)	C11—C13—C14—N5	−1.0 (10)
O5—S2—O6—Cu3	170.8 (5)	C11—C13—C14—C15	179.8 (8)
O6—S2—O5—Cu2	−177.9 (4)	C12—C11—C13—C14	−178.9 (10)
O7—S2—O5—Cu2	−58.5 (6)	C14—N5—N6—C11	−1.9 (8)
O7—S2—O6—Cu3	51.8 (7)	C16—C18—C19—N7	0.6 (9)
O8—S2—O5—Cu2	62.5 (5)	C16—C18—C19—C20	−179.9 (8)
O8—S2—O6—Cu3	−68.5 (7)	C17—C16—C18—C19	−179.0 (9)
O9—S3—O10—Cu4	166.2 (6)	C19—N7—N8—C16	0.2 (8)
O10—S3—O9—Cu3	177.9 (5)	C21—C23—C24—N9	0.9 (9)
O11—S3—O9—Cu3	−62.7 (7)	C21—C23—C24—C25	−179.6 (7)
O11—S3—O10—Cu4	47.1 (7)	C22—C21—C23—C24	176.1 (8)
O12—S3—O9—Cu3	58.7 (7)	C24—N9—N10—C21	−1.7 (8)
O12—S3—O10—Cu4	−73.3 (8)	C26—C28—C29—N11	0.1 (9)
O13—S4—O14—Cu1^ii^	163.1 (6)	C26—C28—C29—C30	179.3 (8)
O13—S4—O14—Cu1^i^	−9.3 (4)	C27—C26—C28—C29	−179.5 (10)
O14—S4—O13—Cu4	178.2 (4)	C29—N11—N12—C26	1.2 (8)
O15—S4—O13—Cu4	57.9 (5)	C31—C33—C34—N13	−0.1 (9)
O15—S4—O14—Cu1^ii^	−75.2 (8)	C31—C33—C34—C35	−177.6 (7)
O15—S4—O14—Cu1^i^	112.4 (6)	C32—C31—C33—C34	177.3 (8)
O16—S4—O13—Cu4	−62.9 (5)	C34—N13—N14—C31	−1.5 (8)
O16—S4—O14—Cu1^i^	−127.3 (6)	C36—C38—C39—N15	−0.4 (10)
O16—S4—O14—Cu1^ii^	45.1 (8)	C36—C38—C39—C40	−179.9 (8)
N1—N2—C1—C2	−178.3 (7)	C37—C36—C38—C39	179.1 (9)
N1—N2—C1—C3	0.5 (8)	C39—N15—N16—C36	−1.8 (8)
N2—N1—C4—C3	1.0 (8)	C41—C43—C44—N17	0.3 (9)
N2—N1—C4—C5	179.1 (7)	C41—C43—C44—C45	−179.8 (8)
N2—C1—C3—C4	0.1 (9)	C42—C41—C43—C44	177.7 (9)
N3—N4—C6—C7	−176.6 (6)	C44—N17—N18—C41	−0.2 (8)
N3—N4—C6—C8	0.8 (8)	C46—C48—C49—N19	−1.3 (9)
N4—N3—C9—C8	0.0 (8)	C46—C48—C49—C50	−178.5 (8)
N4—N3—C9—C10	−179.9 (6)	C47—C46—C48—C49	177.9 (8)
N4—C6—C8—C9	−0.8 (9)	C49—N19—N20—C46	−2.0 (8)
N5—N6—C11—C12	−179.9 (8)	C51—C53—C54—N21	1.9 (9)
N5—N6—C11—C13	1.3 (9)	C51—C53—C54—C55	−177.0 (7)
N6—N5—C14—C13	1.7 (8)	C52—C51—C53—C54	175.2 (8)
N6—N5—C14—C15	−179.1 (7)	C54—N21—N22—C51	−0.8 (8)
N6—C11—C13—C14	−0.2 (10)	C56—C58—C59—N23	0.5 (10)
N7—N8—C16—C17	178.9 (7)	C56—C58—C59—C60	−178.2 (8)
N7—N8—C16—C18	0.1 (8)	C57—C56—C58—C59	177.0 (9)
N8—N7—C19—C18	−0.5 (8)	C59—N23—N24—C56	−1.2 (8)
N8—N7—C19—C20	180.0 (7)	C61—C63—C64—N25	−0.6 (9)
N8—C16—C18—C19	−0.4 (9)	C61—C63—C64—C65	−179.8 (9)
N9—N10—C21—C22	−176.0 (6)	C62—C61—C63—C64	−177.9 (9)
N9—N10—C21—C23	2.3 (9)	C64—N25—N26—C61	−1.2 (8)
N10—N9—C24—C23	0.4 (8)	C66—C68—C69—N27	0.9 (9)
N10—N9—C24—C25	−179.1 (7)	C66—C68—C69—C70	−177.7 (7)
N10—C21—C23—C24	−1.9 (9)	C67—C66—C68—C69	174.9 (8)
N11—N12—C26—C27	179.0 (8)	C69—N27—N28—C66	−1.4 (7)
N11—N12—C26—C28	−1.1 (9)	C71—N29—N30—C74	−0.4 (8)
N12—N11—C29—C28	−0.7 (8)	C71—C73—C74—N30	−1.1 (9)
N12—N11—C29—C30	180.0 (7)	C71—C73—C74—C75	−179.4 (9)
N12—C26—C28—C29	0.6 (9)	C72—C71—C73—C74	−178.9 (8)
N13—N14—C31—C32	−176.9 (7)	C76—C78—C79—N31	−0.8 (9)
N13—N14—C31—C33	1.4 (8)	C76—C78—C79—C80	178.6 (8)
N14—N13—C34—C33	0.9 (8)	C77—C76—C78—C79	−177.9 (9)
N14—N13—C34—C35	178.5 (7)	C79—N31—N32—C76	−0.6 (8)
N14—C31—C33—C34	−0.8 (9)		

Symmetry codes: (i) *x*, *y*−1, *z*; (ii) *x*, *y*+1, *z*.

### Hydrogen-bond geometry (Å, º)

**Table:**

*D*—H···*A*	*D*—H	H···*A*	*D*···*A*	*D*—H···*A*
N2—H2···O4	0.86	2.08	2.792 (7)	139
N6—H6···O3	0.86	2.04	2.889 (7)	168
N10—H10···O3	0.86	2.11	2.869 (7)	146
N12—H12···O4	0.86	2.12	2.951 (8)	163
N14—H14···O8	0.86	2.10	2.835 (7)	143
N16—H16···O5	0.86	2.44	2.889 (7)	114
N16—H16···O7	0.86	2.04	2.894 (8)	173
N18—H18···O6	0.86	2.39	2.866 (8)	116
N18—H18···O8	0.86	2.14	2.988 (7)	169
N20—H20···S3	0.86	2.76	3.504 (6)	146
N20—H20···O9	0.86	2.41	2.885 (9)	115
N20—H20···O11	0.86	2.08	2.933 (7)	171
N22—H22···O7	0.86	2.05	2.828 (7)	150
N24—H24···O12	0.86	2.16	2.840 (8)	135
N26—H26···O16	0.86	2.02	2.875 (7)	171
N28—H28···O15	0.86	2.07	2.803 (7)	143
N30—H30···O10	0.86	2.31	2.817 (8)	118
N30—H30···O12	0.86	2.24	3.083 (8)	165
N32—H32···O11	0.86	2.12	2.857 (7)	144
C30—H30*C*···O5	0.96	2.39	3.213 (11)	144
C50—H50*A*···O6	0.96	2.23	3.124 (9)	155
C65—H65*B*···O10	0.96	2.27	3.192 (11)	160
C70—H70*B*···O10	0.96	2.35	3.116 (10)	137
C2—H2*A*···N16^iii^	0.96	3.01	3.722 (10)	132
C2—H2*A*···O7^iii^	0.96	28	3.806 (9)	146
C53—H53···N8^iv^	0.93	3.07	3.66 (1)	123
C32—H32*B*···N32^v^	0.96	3.00	3.792 (10)	140
C32—H32*B*···N31^v^	0.96	3.17	3.984 (10)	143
C32—H32*B*···N28^v^	0.96	2.87	3.735 (11)	150

Symmetry codes: (iii) *x*+1/2, −*y*+1/2, *z*; (iv) −*x*+1/2, *y*+1/2, *z*+1/2; (v) −*x*+1/2, *y*−1/2, *z*+1/2.
